# Chemical Diversity and Prediction of Potential Cultivation Areas of *Cistanche* Herbs

**DOI:** 10.1038/s41598-019-56379-x

**Published:** 2019-12-24

**Authors:** Ye Wang, Li Zhang, Zhixia Du, Jin Pei, Linfang Huang

**Affiliations:** 1 Institute of Medicinal Plant Development, Chinese Academy of Medical Sciences, Peking Union Medical College, Engineering Research Center of Chinese Medicine Resource, Ministry of Education, Key Research Laboratory of Traditional Chinese Medicine Resources Protection, Administration of Traditional Chinese Medicine, National Administration of Traditional Chinese Medicine, Beijing, 100193 China; 2 College of Science, Sichuan Agricultural University, Ya’an, 625000 China; 3 College Pharmacy, Chengdu University of Traditional Chinese Medicine, Chengdu, 611137 China

**Keywords:** Biochemistry, Plant sciences, Biogeochemistry, Environmental sciences

## Abstract

Owing to hostile growth environments and increasing related production, *Cistanche* plants have decreased in number. The aim of the present study was to evaluate the quality of and to predict potential suitable regions for two official species and two nonofficial species (*C. salsa* and *C. sinensis*) by high-performance liquid chromatography and the MaxEnt model. The results indicated that 2′-acetylacteoside was present only in *C. deserticola*. The compound can be used as a potential chemical marker to discriminate *C. deserticola* from the three other *Cistanche* plants. Anthocyanin A and carotenoid F were the common constituents of the two official species only and can thus be used as chemical markers to differentiate between official and nonofficial species. The prediction results of a potentially suitable distribution indicated that *C. sinensis* has much wider regions for potential distribution than the other species. Finally, the echinacoside content in *C. deserticola* was significantly different between the two suitable potential distributions, and the contents of samples from Inner Mongolia were significantly higher than those from Gansu Province. This is the first application of the combination of the contents of chemical components and the results of MaxEnt models for the quality assessment of herbal medicine. Our results may provide a reference for the sustainable utilization of endangered *Cistanche* species.

## Introduction

Since the 72nd World Health Assembly, traditional medicine has been included in the International Classification of Diseases (11th version), which mainly dates from traditional Chinese medicine and is also widely accepted in Southeast Asian countries such as Japan, Korea, and India. The conference indicated that traditional medicine plays an important role in health regulation. Medicinal herbs are the material base of traditional medicine in addition to acupuncture and manipulation. The accurate usage and combination of different herbs determine the ultimate efficacy of traditional medicine^1–3^. In addition, the content and type of active components in herbs fluctuate with their botanical origins and growth environments. Therefore, proper botanical origins and suitable growth environments are vital for clinical efficacy and security.

*Cistanche* plants are utilized as a precious tonic and edible herbs for men’s care in China, Japan and some Southeast Asian countries^4,5^. Four species of the genus are known for their potential medicinal value in China, namely, *C. deserticola*, *C. tubulosa*, *C. salsa*, and *C. sinensis*. The dried succulent stems of the first two species are recorded in the Chinese Pharmacopoeia (2015 edition), named Cistanches Herba. These species are beneficial to the kidneys and intestinal tract. The other two species are consumed in some locations or are utilized as adulterants of Cistanches Herba. *C. salsa* was recorded in the local herbal standards in Gansu (1992 edition) and Xinjiang (1987 edition) provinces. *C. sinensis* is a unique species of China. Modern chemical and pharmacological studies have shown that chemical components from these herbs have several effects, such as brain function improvement, aphrodisiac effects, and immune-boosting effects^6^.

Published studies indicate the content and type difference of chemical constituents among different *Cistanche* plants, in which betaine, Krebs cycle intermediates, phenylethanoid glycosides and iridoids were regarded as four chemical markers responsible for discrimination analysis between *C. deserticola* and *C. tubulosa*^7,8^. A detailed study indicated that the isomers of campneoside II, cistanoside C, and cistanoside A were three potential chemical markers to distinguish the two species mentioned above^9^. Further research showed that eight phenylethanoid glycosides could be chosen as chemical markers for discriminating the *Cistanche* species, which mainly include *C. deserticola*, *C. tubulosa* and *C. sinensis*^10^. In addition, the contents of chemical constituents vary with geographical origin. Zhou and his coauthors indicated that the total contents of seven index components (castanoside A, echinacoside, isoacteoside, 2′-actylacteoside, castanoside C, and tubluoside B) of *C. tubulosa* from south of Xinjiang were approximately six times those of Kuitun and Hami in China^11^. Among these chemical components, acteoside and echinacoside are regarded as index components for the quality control of herbal medicine. The two components have been reported to improve brain function, and acteoside mainly contributes to the aphrodisiac effect^6^.

Species distribution models (SDMs) are statistical models established with existing environmental variables to infer species’ ecological requirements. Such models can map a target species’ potential distributions on the basis of the observed distributional data^12^. To date, SDMs have been successfully applied to the prediction of the distribution tendency of endangered and ecological plants during climate change^13,14^. Among these models, the MaxEnt model is commonly used as a simple means of predicting the habitat suitability distribution with presence-only data and performs well with incomplete data, small sample sizes and gaps^14^. Combined with some software (such as ArcGIS), research can extract environmental and climate variables of collection sites of samples. Furthermore, a potentially suitable location can be divided into three or four levels^15–17^. However, no study has reported combined strategies that incorporate potential suitable locations after division and chemical component contents for the quality control of herbal medicine.

In the present study, four endangered *Cistanche* plants were collected for quality assessment with high-performance liquid chromatography (HPLC), and seven chemical components were used as indices. Furthermore, a MaxEnt model equipped with 26 environmental variables was utilized to predict potentially suitable areas for investigating optimal species for further usage. Finally, the combination of the index component contents and suitable areas was systematically analyzed for better development and utilization of these endangered *Cistanche* plants in China and other countries.

## Results

### Chromatographic analyses

The contents of the seven chemical components were calculated on the basis of their calibration curves. The seven equations of the calibration curves were Y = 20.5400X+0.1616 (2′-acetylacteoside, R = 0.9992), Y = 18.5640X+0.3724 (acteoside, R = 0.9821), Y = 15.6350X+0.0090 (isoacteoside, R = 0.9999), Y = 12.7460X+0.0103 (tubuloside A, R = 0.9999), Y = 10.638X+0.1615 (cistanoside A, R = 0.9997), Y = 21.533X−0.0006 (cistanoside F, R = 1), and Y = 13.0740X+1.1381 (echinacoside, R = 0.9821); the concentration (X) is the horizontal axis, and the peak area (Y) is the vertical axis. R is the correlation coefficient and indicates an excellent linear correlation between these calibration curves. A precision experiment (six sequential injections of the same sample extractives) showed that the precision was good, and the relative standard deviation (RSD) of the peak area was between 0.3% and 0.8%. Similarly, the repeatability (six sequential injections of the same sample extractives) and stability tests (six injections of the same sample extractives after 0, 2, 4, 8, 12, and 24 h) yielded RSDs of 0.56–1.46% and 1.06–3.60%, respectively.

Further content analyses of the seven index constituents were displayed in grouped horizontal boxes containing visual comparison results and significant differences in lowercase. The recovery test results of the seven constituents varied from 99.25% to 104.10% with RSDs between 0.69% and 3.45%. Therefore, the method was accurate.

**Figure 1:**
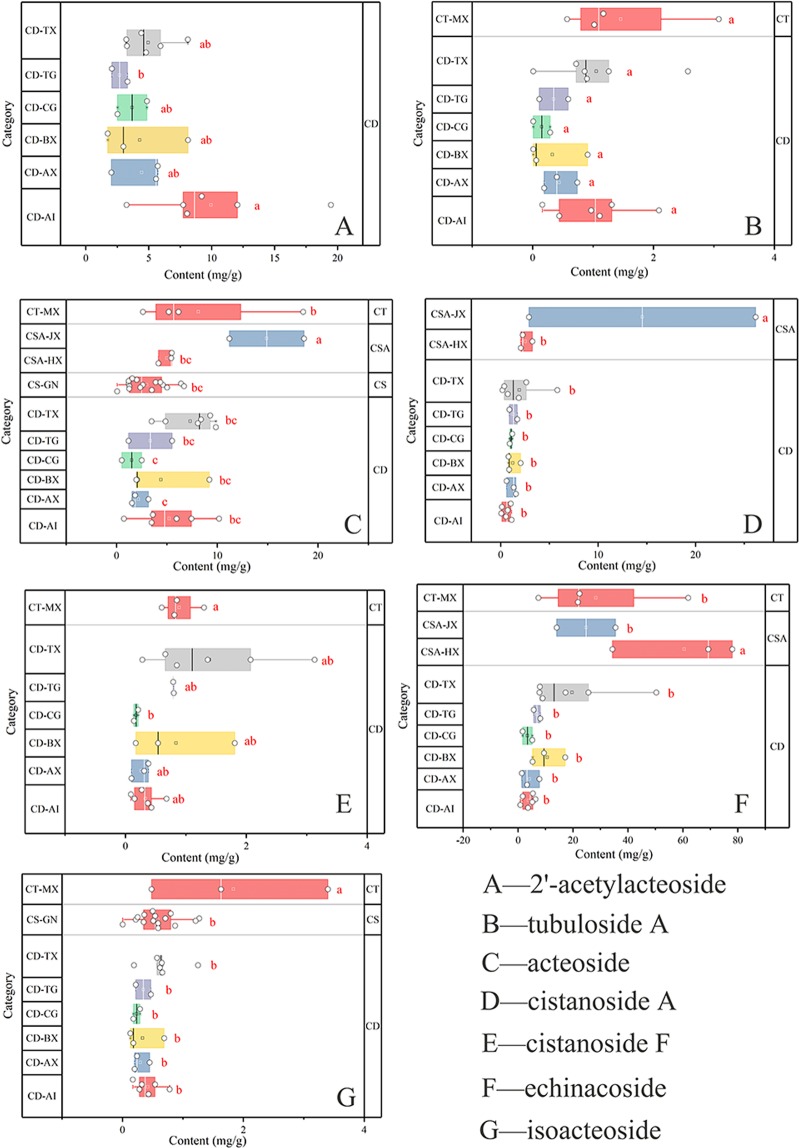
Contents of seven chemical components in the four *Cistanche* species.

The contents of the seven index constituents in the four species and the geographical origins of the same species are displayed in grouped horizontal boxes. The contents of some chemical components were under the detection limit. Thus, only a portion of the figures contained four *Cistanche* species. The chemical component 2′-acetylacteoside was detected only in *C. deserticola*, whereas it was undetected in the other species by the present method. However, a content difference was observed among the various geographical origins focused on 2′-acetylacteoside. The contents of the component from Alashanzuoqi in Inner Mongolia were significantly higher than those in the three other provinces, whereas the contents in the species from Tingtuhu of Minqinin, Gansu Province, were significantly lower than those in the other locations (Fig. 1A). Tubuloside A was detected in *C. deserticola* and *C. tubulosa*. No remarkable difference was observed between the two species and even among plants of the same species from different geographical origins (Fig. 1B). The four species contained the same chemical component (acteoside). The content of acteoside in *C. salsa* collected from Jianga’erhan of Tacheng in Xinjiang was significantly higher than that of the other species and the same species from different growing areas. In contrast, the content of acteoside did not differ significantly among the various origins of *C. deserticola* (this finding was the same as that for tubuloside A; Fig. 1C). Cistanoside A was detected in *C. deserticola* and *C. salsa*. The content of the *C. salsa* samples from Jianga’erhan of Tacheng in Xinjiang was significantly higher than that of *C. deserticola* samples from other places. Moreover, the variation in the location did not affect the content of the component. This condition was the same as that for tubuloside A and acteoside (Fig. 1D). For cistanoside A, the content of the index component did not fluctuate with the variations in species and geographical origins (Fig. 1E). The echinacoside content of the *C. salsa* samples from Hejiaoke of Tuoli in Xinjiang was significantly higher than the content of the samples from Jianga’erhan of Tacheng in Xinjiang. The component contents of the samples from *C. deserticola* and *C. tubulosa*, regardless of where they were collected, were significantly lower than in other locations (Jianga’erhan of Tacheng in Xinjiang). In addition, the chemical component could not be detected in *C. sinensis* (Fig. 1F). Isoacteoside was detected in all *Cistanche* species except *C. salsa*; the isoacteoside content in *C. tubulosa* was significantly higher than that in the two other species regardless of the source provinces in China (Fig. 1G). Herein, all of the significant differences were less than *P* < 0.5.

**Figure 2:**
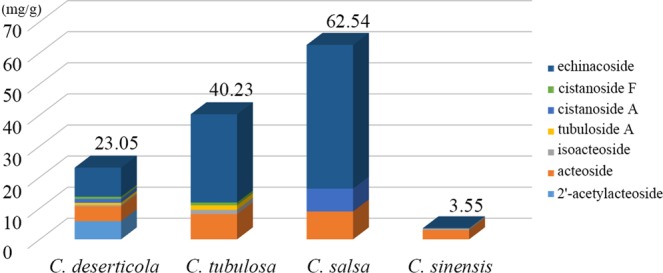
Total content of styrene glycosides in the four *Cistanche* species.

The total contents of these chemical components are stacked in Fig. 2 for an improved comparison of the seven styrene glycosides. Herein, we hypothesized that geographical origins exerted a small influence on the accumulation of these chemical components compared with species differences. Seven types of styrene glycosides were detected in the *C. deserticola* samples. However, the total content was lower than that of *C. tubulosa* and *C. salsa*. The results indicated that *C. salsa* had the highest content of styrene glycosides although only echinacoside, castanoside A and acteoside were detected by liquid chromatography. *C. sinensis* contained only isoacteoside and acteoside and thus had the lowest content of total styrene glycosides. According to the recorded standard in the China Pharmacopoeia, the contents of echinacoside and acteoside in *C. deserticola* should be higher than 3 mg/g, and those in *C. tubulosa* should be higher than 15 mg/g. The detection results indicated that most of the *C. deserticola* samples conformed to the standards except for samples from Changcheng, Liangzhou in Gansu Province (acteoside content was 1.51 mg/g) and Aibi Lake region, Tacheng in Xinjiang Province (acteoside content was 2.18 mg/g), whose contents were lower than the standards. In addition, the acteoside content in *C. tubulosa* (8.1225 mg/g) was lower than the standards, whereas the content of echinacoside was higher than the standard (28.38 mg/g).

### Potential suitable areas in the world and in china

With the combined calculations from MaxEnt and ArcGIS, the potential suitable areas for the four *Cistanche* species of herbs were calculated. The results are shown in Table 1. Similar to the categories in the above sections, the potential suitable areas were divided into four classes. Regarding the high habitat suitability class, three species had approximately 30 km^2^ larger suitable areas worldwide (*C. deserticola*: 29.2210 km^2^, *C. sinensis*: 31.3034 km^2^, *C. salsa*: 30.8633 km^2^) compared with *C. tubulosa* (10.6866 km^2^). As shown by the comparison of the high habitat suitability class, *C. deserticola* had the largest acreage (38.9267 km^2^) in terms of the moderately suitable class (*C. tubulosa*: 12.4069 km2, *C. sinensis*: 32.1292 km^2^, *C. salsa*: 38.5764 km^2^), while the area of *C. tubulosa* was still the smallest.

**Table 1:** The potential suitable areas of four *Cistanche* species herbs.

	High habitat suitability class (km^2^)	Moderately suitable class (km^2^)	Low suitable class (km^2^)	Not suitable class (km^2^)
*C. deserticola*	29.2210	38.9267	82.7000	21301.2360
*C. tubulosa*	10.6866	12.4069	48.7369	21380.2520
*C. salsa*	30.8633	38.5764	83.4967	21299.1460
*C. sinensis*	31.3034	32.1292	58.3883	21336.2620

**Figure 3:**
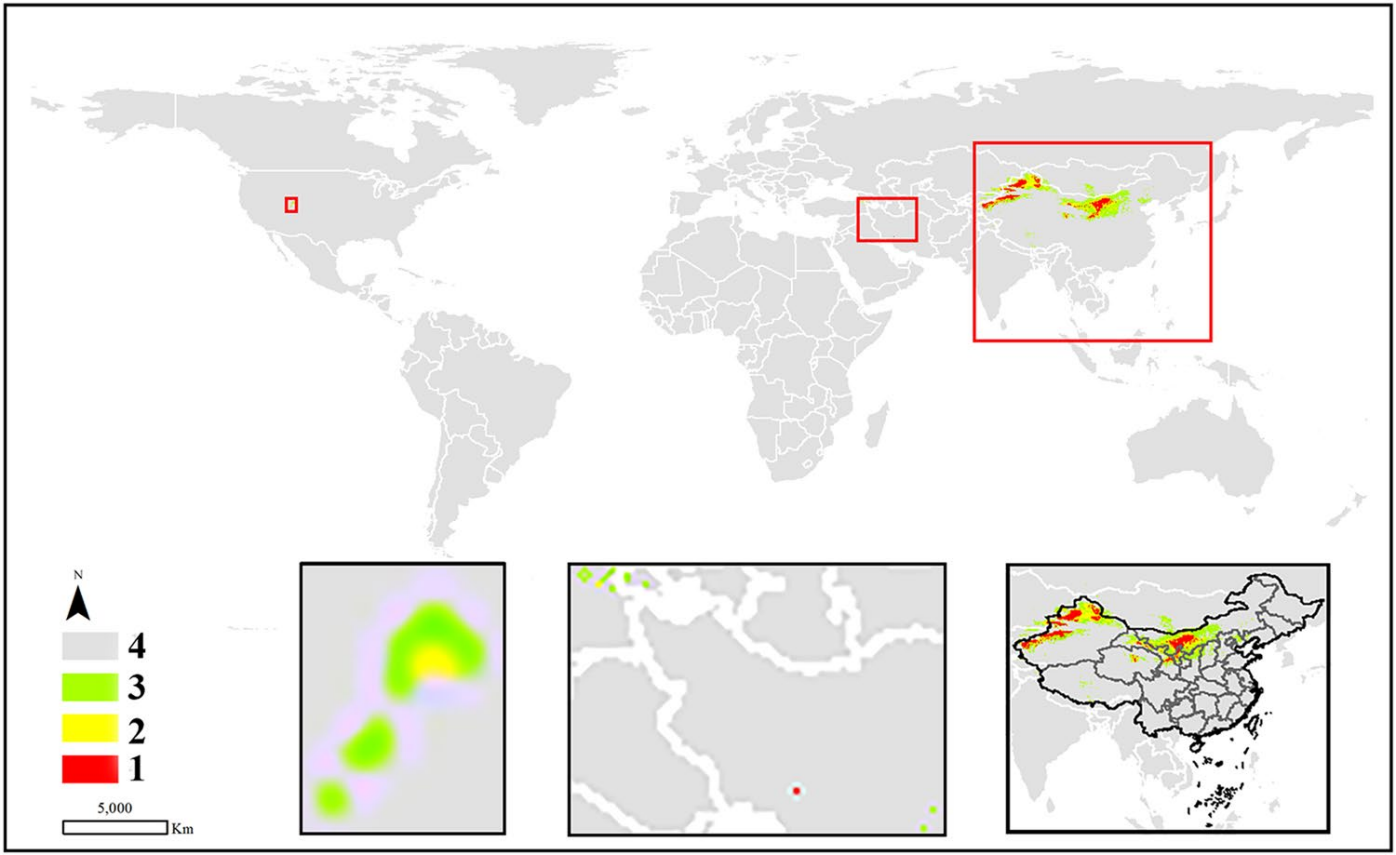
The potential distribution predicted by MaxEnt model in *C. deserticola*. (1: high habitat suitability class, 2: moderately suitable class, 3: low suitable class 4: very low suitable class or not suitable class. The figure was accomplished by ArcGIS (version 10.0) and MaxEnt (version 3.4.1) software http://www.cs.princeton.edu/schapire/maxent).

**Figure 4:**
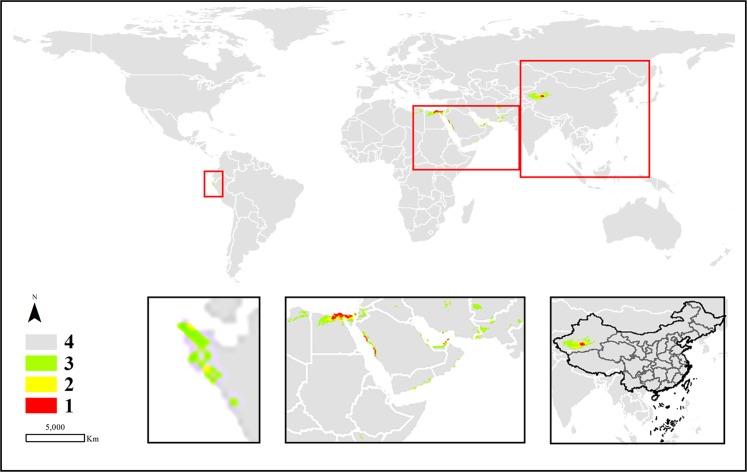
The potential distribution predicted by MaxEnt model in *C. tubulosa*. (1: high habitat suitability class, 2: moderately suitable class, 3: low suitable class 4: very low suitable class or not suitable class. The figure was accomplished by ArcGIS (version 10.0) and MaxEnt (version 3.4.1) software http://www.cs.princeton.edu/schapire/maxent).

**Figure 5:**
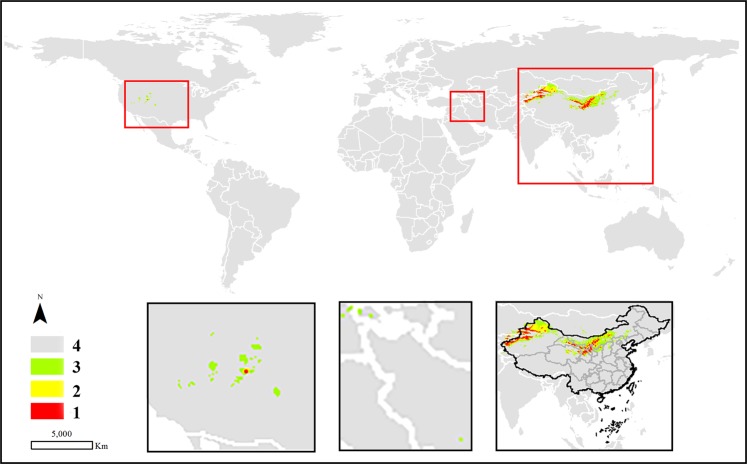
The potential distribution predicted by MaxEnt model in *C. salsa*. (1: high habitat suitability class, 2: moderately suitable class, 3: low suitable class 4: very low suitable class or not suitable class. The figure was accomplished by ArcGIS (version 10.0) and MaxEnt (version 3.4.1) software http://www.cs.princeton.edu/schapire/maxent).

**Figure 6:**
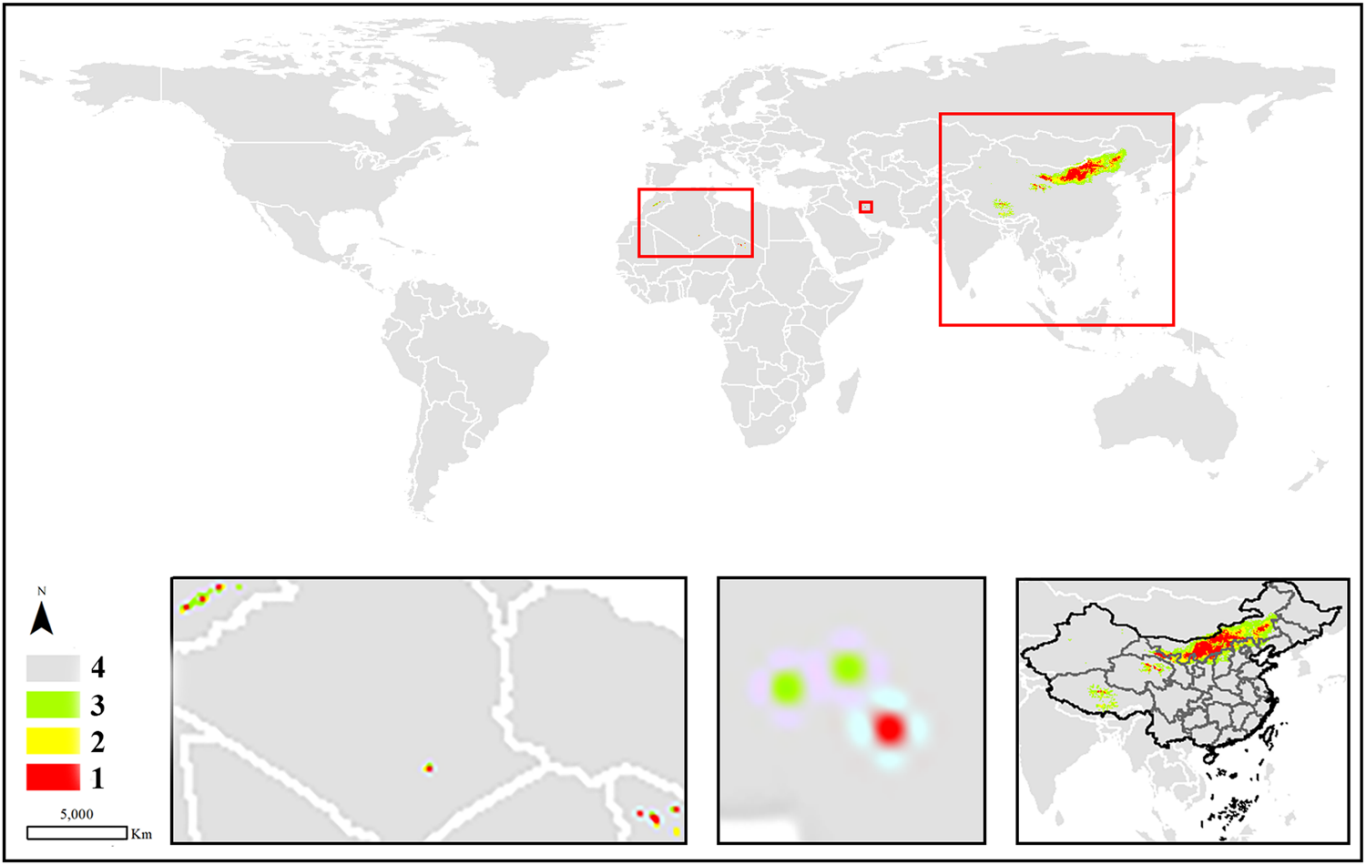
The potential distribution predicted by MaxEnt model in *C. sinensis*. (1: high habitat suitability class, 2: moderately suitable class, 3: low suitable class 4: very low suitable class or not suitable class. The figure was accomplished by ArcGIS (version 10.0) and MaxEnt (version 3.4.1) software http://www.cs.princeton.edu/schapire/maxent).

In terms of the potential areas regarded as potential distribution or suitable cultivation locations, the high habitat suitability and moderately suitable classes are discussed as follows. Most of the suitable areas (approximately 90%) for *C. deserticola* were mainly distributed in the north and northwest of China. In addition, a few areas with potential cultivation conditions were identified in the south of the United States, southwestern Iran, eastern Turkey, southern Mongolia, and eastern Kazakhstan (Fig. 3). Unlike the abovementioned species, *C. tubulosa* seemed to be well distributed worldwide, covering the west of China, northwest of South America, north of Africa, and some countries between China and Africa. However, although *C. tubulosa* had a wide potential distribution, the total area of the species was the smallest compared with the other three species, for which the total areas of the high habitat suitability and moderate classes were 23.0935 km^2^. Regarding the two classes, only a few potential areas suitable for the growth of the species were found in northern Egypt, in the western region of the Kingdom of Saudi Arabia, southern Yemen, northern United Arab Emirates, southern Iran, and Pakistan. The largest areas were still in China, where the total prediction areas were located in the west of the country (Fig. 4). Similar to *C. deserticola*, *C. salsa* was mainly distributed in the northwest and north of China, where the specimen has records. After prediction by the MaxEnt model, there was a small prediction area that may be a suitable growth environment in the United States and eastern of Kazakhstan and Kyrghyzstan (Fig. 5). Most of the predicted locations of *C. sinensis* were distributed in northern and western China. Additionally, a few areas were identified in the middle part of Morocco, southeastern Algeria, and northwestern Chad and Iran (Fig. 6).

The potentially suitable areas calculated by MaxEnt are displayed in the bottom right corner of Figs. 3–6 to show the prediction distribution of the four species in China. Herein, the smallest units of prediction distribution mapping were based on the provinces in China. Similar to the analysis for the world, the analysis in this section was based on high habitat suitability and moderate classes. The prediction areas indicated that *C. deserticola* was mainly distributed in Xinjiang, Inner Mongolia, Ningxia, Gansu, and Qinghai provinces. Although a few distribution areas were found in other provinces, they belonged to Class 2. Therefore, these regions were poorly suitable and thus not recommended for the cultivation of the species. For *C. tubulosa*, all potentially suitable regions of the species were predicted in Xinjiang Province in China. Interestingly, *C. salsa* had a wide range of potential distributions in China, including Xinjiang, Inner Mongolia, Ningxia, Gansu, Qinghai, Shanxi, Shaanxi and Hebei provinces. Moreover, the entire Ningxia Province seemed to be a possible cultivation area for the species. Similar to *C. salsa*, *C. sinensis* also had wide regions for potential distribution excluding Xinjiang Province (but it had a prediction area in Xizang Province).

### Contribution of environmental variables

With the help of two indices (percent contribution and permutation importance in the.html file), these bioclimatic variables were ranked based on their importance for the MaxEnt model. Thirty-nine important variables contributed to the *C. deserticola* model, of which srad08, tmax12, pre01, and tmax02 were the most important variables; that is, their contribution percentages exceeded 10% (Table S1). For *C. tubulosa*, twenty-nine variables contributed to the model, and pre09, srad05, pre08, and vapr08 were the vital environmental factors (Table S2). Thirty variables were contributing variables for the *C. salsa* model, and tmax02, srad08, bio19, and pre01 were vital factors, with contribution percentages exceeding 10% (Table S3). The *C. sinensis* model had 35% contribution variables, among which tmax02, bio19, and pre12 were the most important (Table S4).

Furthermore, a jackknife test was performed to select the vital bioclimatic variables, which may differ from the aforementioned variables. In general, three plots were used to show variable importance, namely, regularized train gain, test gain, and AUC of the test data. The results of the jackknife test for the *C. deserticola* model are shown in Fig. S1. Herein, srad07, bio09, and wind10 provided very high gain when used independently or omitted, indicating that srad07 contained more useful information by itself than the other variables did. By contrast, bio09 and wind10, which were not present in the other variables, appeared to have the most information. Unlike the species, srad05, pre10, and tmax07 were the vital variables for *C. tubulosa* and provided high gains for the model. The other variables providing the gain value are displayed in Fig. S2. The gain results of the *C. salsa* model are shown in Fig. S3. These plots indicate that four variables provided higher gains for the model, namely, srad11, srad06, bio09 and pre02. Regarding the MaxEnt model and the potential distribution of *C. sinensis*, four bioclimatic variables, srad11, srad12, win08, and tmax11, were deemed important variables; they contained more useful information by themselves than the other variables did (Fig. S4). Therefore, the abovementioned variables contained the most important information contributing to the model gain. Interestingly, a few differences were identified between the results of the jackknife test and the permutation importance (or percentage importance). Three variables (srad07, srad11 and tmax07) were important variables with high gains in the results of the jackknife test compared with the two other methods.

### Evaluation of each species model

**Figure 7:**
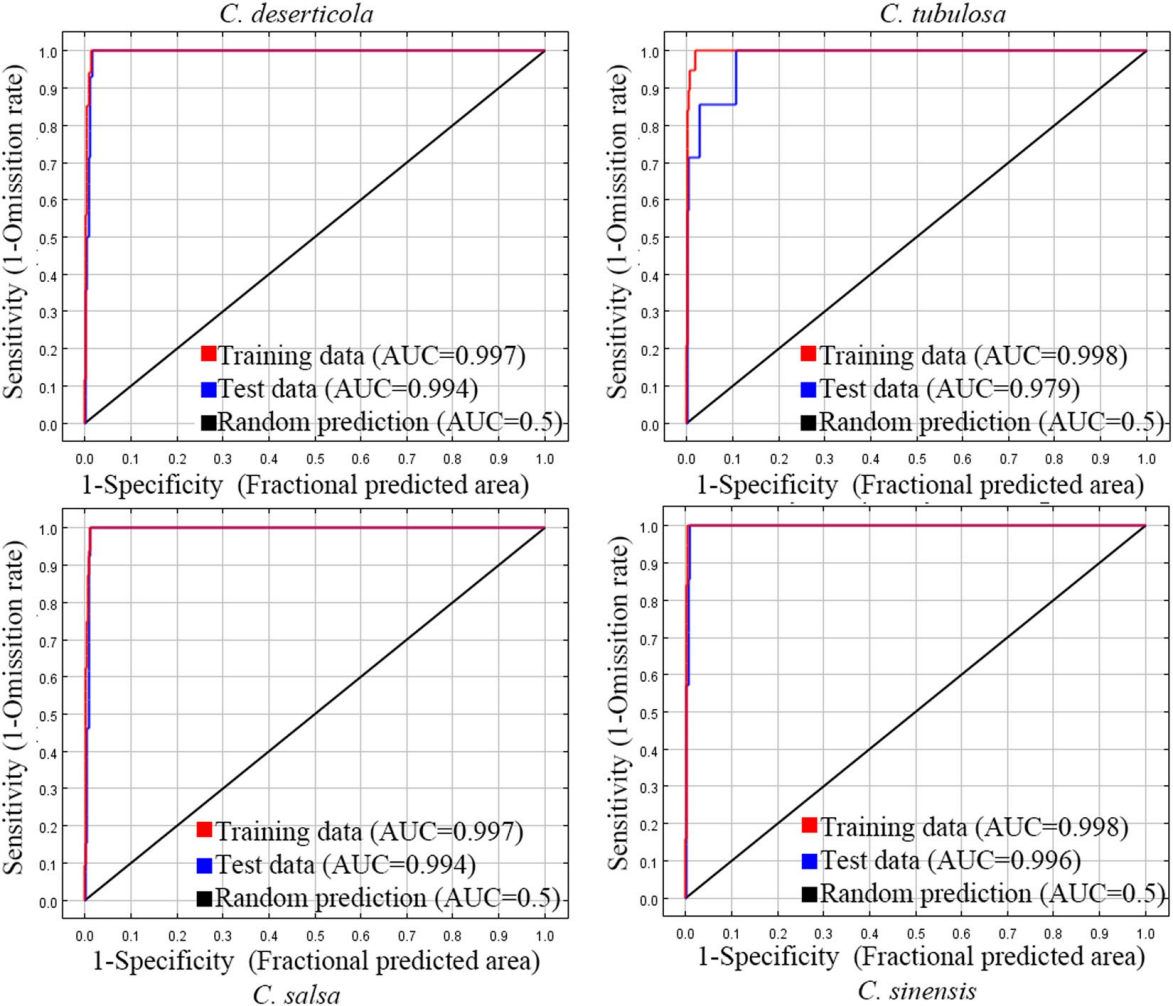
ROC curves of MaxEnt models for four *Cistanche* species.

Model performance was divided into five categories: fail, poor, fair, good, and excellent (0.9 < AUC ≤ 1). The model performance in terms of the AUC value and ROC curve among the four models is displayed in Fig. 7. The results indicated that the four models’ performance in the training data was excellent, with AUC values between 0.997 and 0.998 and a top left corner ROC curve close to 1. Moreover, the AUC value in the test data also showed good model performance, with a high value between 0.979 and 0.996 and a top left corner ROC curve close to 1. In general, these models have a strong capability to predict potential species distribution.

### Variables’ response analysis to suitability

Response curves show the quantitative relationship between habitat suitability and environmental variables (also known as the logistic probability of presence)^14^. In the present study, the common variables between the jackknife test and permutation importance (percentage contribution) were used to analyze the variables’ response to suitability. In terms of *C. deserticola*, bio09 and wind10 were common variables. The suitable mean temperature of the driest quarter (bio09) was between −12.71227957 °C and −1.296478534 °C. The optimal wind speed in October (wind10), which is beneficial for the distribution of the species, was between 0.5841 and 3.2581 m·s^−1^ (Fig. S5). Two common variables were identified for the potential distribution in the *C. tubulosa* model. The first one was solar radiation in May, with a suitable range exceeding 24661.0800 kJ·m^−2^·day^−1^. The other was precipitation in October (pre10), with an optimal value lower than 5.4640 mm (Fig. S6). The results of the jackknife test showed that four variables were the same as those of the two other evaluation methods for the contribution of the bioclimatic factors in the *C. salsa* model. The first two variables comprised solar radiation in June and November (srad06 and srad11), with ranges of 23298.9691–25137.457 and 6709.6219–10687.2852 kJ·m^−2^·day^−1^, respectively. The third was the mean temperature of the driest quarter (bio09), with an optimal range of -12.6159–2.8479 °C. The fourth variable was precipitation in September (pre09), with a suitable range of 8.3762–32.4312 mm (Fig. S7). The MaxEnt model of *C. sinensis* had three important variables that mostly contributed to the potential distribution of the species. The suitable range of solar radiation in December (srad12) was 6138.3161–9488.8316 kJ·m^−2^·day^−1^, and that of maximum temperature in November (tmax11) was -1.9115–9.6048 °C. Furthermore, the suitable range of wind speed in August (wind08) was 2.1005–3.3054 m·s^−1^ (Fig. S8). The other important variables contributing to the potential distribution of the four species are summarized in Figs. S5–S8.

### Correlation between chemical components and bioclimatic variables

**Figure 8:**
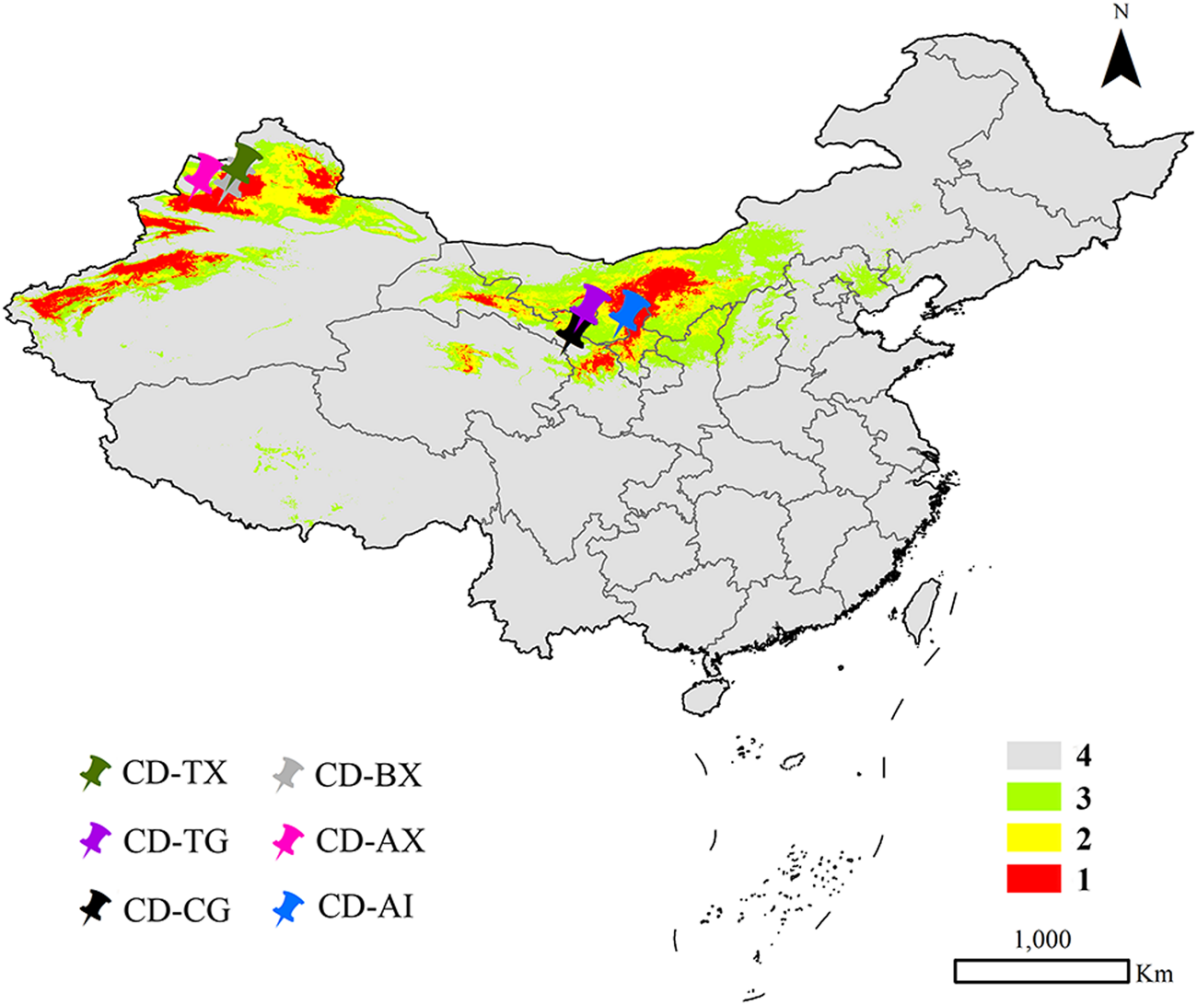
Location in China map of collected *C. deserticola* samples. (1: high habitat suitability class, 2: moderately suitable class, 3: low suitable class 4: very low suitable class or not suitable class. The figure was accomplished by ArcGIS (version 10.0) and MaxEnt (version 3.4.1) software http://www.cs.princeton.edu/schapire/maxent).

The most popular species from the main distribution provinces (*C. deserticola*) was compared to finish the correlation analysis and to investigate the content fluctuation of seven index components with the environmental condition. After analysis of the suitable potential distribution of the species, these potential distribution regions were divided into four categories. Herein, six collection sites including 22 samples were mapped on the China map extracted from the results of the MaxEnt model of the species (Fig. 8). The results indicated that two collection sites were situated in Class 1 (high habitat suitability class), and two collection locations belonged to Class 2 (moderately suitable class). The two final sites were classified as Class 3 (low suitable class). A significant difference in the 2′-acetylacteoside content was observed between CD-TG (Tingtuhu, Minqin, Gansu Province) and CD-AI (Alashanzuoqi, Inner Mongolia), which belonged to Classes 2 and 1, respectively. In addition, the content of the component in CD-AX (Aibi Lake region, Tacheng, Xinjiang) and CD-CG (Changcheng, Liangzhou, Gansu Province), which belonged to Class 3, did not significantly differ from those of the four other sites, which belonged to Class 1 and Class 2. For the six other chemical components, no significant difference was identified among the different geographical origins in terms of the content of these constituents.

## Discussion

Herbal medicine is a complex mixture of many chemical components that contribute to the systematic targeting of disease. In addition, one type of herbal material may be collected from different botanical species that have similar characteristics or effective components. For instance, Coptidis Rhizoma is a herbal medicine with high-frequency usage; it is obtained from three congeneric plants (*Coptis chinensis*, *C. deltoidea* and *C. teeta*) and used to cure diarrhea induced by dampness retention with the help of various berberines^18^. In addition, Polygonatum Rhizoma is commonly utilized as a functional food in daily life in China for immunoregulation; the crude materials are rhizomes of *Polygonatum kingianum*, *P. sibiricum* and *P. cyrtonema*^19^. The present study included four species, two of which are officially recorded in the China Pharmacopoeia (*C. deserticola* and *C. tubulosa* in the 2015 version). The results regarding the chemical components indicated the existence of differences in the type and content among the four species. In particular, echinacoside, carotenoid A, 2′-acetyl verbascoside, anthocyanin, and carotenoid F can be detected in *C. sinensis* and can thus be regarded as an index for determining whether the powder of the official species has been adulterated with nonofficial herbal medicine. 2′-Acetyl verbascoside was only detected in *C. deserticola*, which seems to be the optimal index component indicating that the known herbal materials belong to one of the four species mentioned in our study. These findings are the same as those in our previous study, in which the component was regarded as a potential marker for discriminating *C. deserticola* samples from Xinjiang and Inner Mongolia^20^. Furthermore, anthocyanin A and carotenoid F were common components in the two official species but were not found in the two other species. Therefore, these chemical components may be utilized as chemical markers for the official and nonofficial species for enhanced quality control of crude medicinal materials in the herbal market.

The MaxEnt model established a correlation relationship between environmental variables and potential distribution worldwide for the target species^21,22^. The model has the ability to predict the present distribution and potential suitable regions, which can be regarded as a reference for the cultivation of species, especially endangered ones. In general, *ex situ* conservation is an effective means of protecting endangered species^23^. Protection strategies can ensure normal usage in clinics. In our study, there were potential distribution regions in Qinghai Province in China, southern United States, southwestern Iran, eastern Turkey, southern Mongolia, and eastern Kazakhstan, which were different from the records in Flora Reipublicae Popularis Sinicae (http://frps.iplant.cn/frps/Cistanche%20deserticola) for *C. deserticola*. The predicted regions of *C. tubulosa* are extremely similar to those in the records of Flora Reipublicae Popularis Sinicae (http://frps.iplant.cn/frps/Cistanche%20tubulosa), which mentions that the species are distributed in southern Xinjiang Province in China, northern Africa, Arabian Peninsula, and central Asia. Herein, only a few areas may be suitable for the cultivation of *C. tubulosa* in the southwest of South America. The limited growth regions were confirmed to our actual investigation that the cultivation of the species requires sufficient water. The south of Xinjiang Province has enough water suitable for the wide distribution of the species. The potentially suitable regions of *C. salsa* were different from those in the records, and the predicted regions were larger than the recorded distribution according to http://frps.iplant.cn/frps/Cistanche%20salsa. Similarly, the predicted location of *C. sinensis* slightly differed from that in the records at http://frps.iplant.cn/frps/Cistanche%20sinensis, especially in the middle part of Morocco, southeastern Algeria, and northwestern Chad and Iran, which were identified by the prediction model results to be suitable for the growth of the species. The wide areas of the two latter species implied that herbal farmers can plant the two species to meet the demand of local herbal markets, which mostly depend on wild resources. Our field survey found that collecting wild samples of the two species is becoming increasingly difficult. Approximately 2–3 wild samples existed in 10 quadrats measuring 2 × 2 m in 2012, but the same samples needed 30 of the same quadrats in 2019. These results implied that wild resources are shrinking in quantity. Suitable regions are becoming increasingly limited because of industrial development and road construction. Therefore, based on the potential suitable distribution predicted by the MaxEnt model, researchers should aim to cultivate the two species to enhance the protection of wild samples.

We did not find literature using 12-month minimum temperature, maximum temperature, average temperature, precipitation, solar radiation, wind speed, and water vapor pressure as bioclimatic variables in establishing MaxEnt models. Herein, the six variables (over 12 months), combined with 19 bioclimatic factors, were systematically used to establish the MaxEnt models of the four studied species. The results indicated that the prediction of the potential suitable region of each species depended on different environmental variables. Generally, solar radiation (srad05, srad06, srad12) was the main influential variable among the four species. May and June were interpreted as the flowering and fruiting periods, respectively. Therefore, solar radiation may be the main influential factor that determines the distribution of the four *Cistanche* species.

A combination of high-performance liquid chromatography and the MaxEnt model was used to systematically investigate content fluctuations in seven chemical components. The results indicated variations in constituent type and content among the four *Cistanche* species and among plants of the same species from different geographical origins. Moreover, 2′-acetylacteoside can be utilized as a potential chemical marker for discriminating *C. deserticola* from the three other *Cistanche* plants. Anthocyanin A and carotenoid F were the common constituents of the two official species and can therefore be regarded as a chemical marker to distinguish between official and nonofficial species. The echinacoside content of *C. deserticola* significantly varied between two suitable potential distributions; the contents of the samples from Inner Mongolia were significantly higher than those from Minqin City in Gansu Province. Our results may provide a reference for the enhanced development and utilization of four endangered *Cistanche* species in China and surrounding countries.

## Methods

### Materials

**Figure 9:**
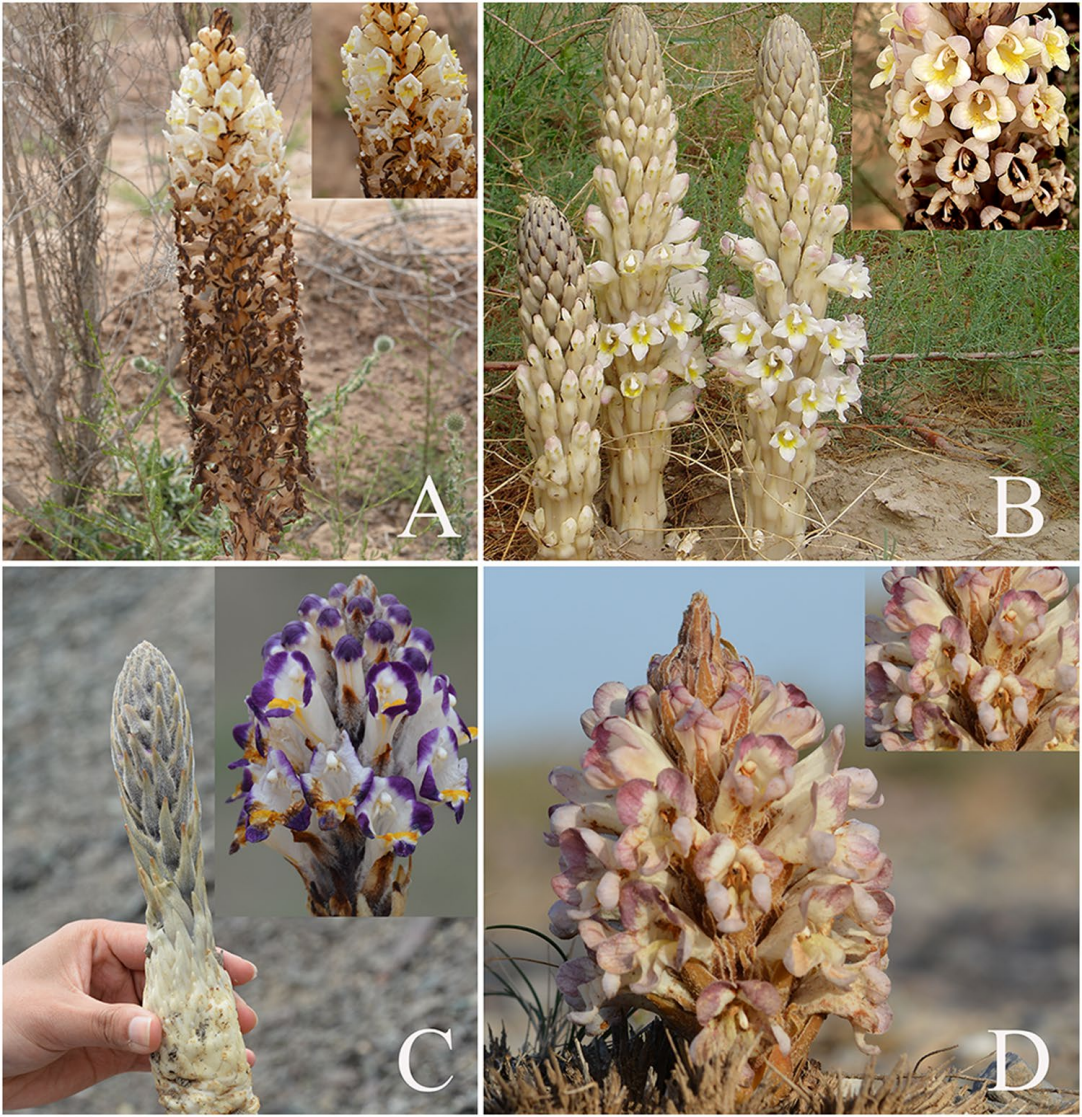
Original plants and amplified inflorescence four *Cistanche* species. (**A**: *C. deserticola*; **B**: *C. tubulosa*; **C**: *C. salsa*; **D**: *C. sinensis*).

A total of 47 wild samples were collected from Xinjiang, Gansu, Ningxia and Inner Mongolia provinces in China. All samples were authenticated as *C. deserticola*, *C. tubulosa*, *C. salsa* and *C. sinensis* belonging to the *Cistanche* genus by Professor Linfang Huang (Institute of Medicinal Plant Development, Chinese Academy of Medical Sciences, Peking Union Medical College). Details about the samples are displayed in Table 2, and the original botanical specimens with their amplified inflorescence are shown in Fig. 9. Fresh samples were washed with tap water and dried under shade. The dried samples were crushed into powder and passed through a 65-mesh sieve. The powder of each sample was stored in Ziploc bags for HPLC analysis.

**Table 2:** Detailed samples information of four *Cistanche* plants.

Codes	Plant name	Geographical origins	Abbreviation of origins
CD1-CD6	*C. deserticola*	Tula, Tacheng, Xinjiang	CD-TX
CD7-CD9	*C. deserticola*	Baijiantan, Tacheng, Xinjiang	CD-BX
CD10-CD11	*C. deserticola*	Tingtuhu, Minqin, Gansu	CD-TG
CD12-CD13	*C. deserticola*	Changcheng, Liangzhou, Gansu	CD-CG
CD14-CD19	*C. deserticola*	Alashanzuoqi, Inner Mongolia	CD-AI
CD20-CD22	*C. deserticola*	Aibi Lake region, Tacheng, Xinjiang	CD-AX
CT1-CT4	*C. tubulosa*	Minfeng, Hetian, Xinjiang	CT-MX
CSA1-CSA3	*C. salsa*	Hejiaoke, Tuoli, Xinjiang	CSA-HX
CSA4-CSA5	*C. salsa*	Jianga’erhan, Tacheng, Xinjiang	CSA-JX
CS1-CS16	*C. sinensis*	Gezi mountain, Qingtongxia, Ningxia	CS-GN

Seven standards (2′-acetylacteoside, acteoside, isoacteoside, tubuloside A, cistanoside A, cistanoside F and echinacoside) of >95% purity were purchased from Chengdu Mansite Biotechnology Co., Ltd. (Chengdu, China) for the qualitative and quantitative analyses of the samples. Methanol, acetonitrile, and ethanol were obtained from Thermo Fisher Scientific (Fair Lawn, NJ, USA). Formic acid (HPLC grade) was obtained from Dikmapure (Lake Forest, CA, USA). Ultrapure water (2.22 μs/cm conductivity) for the entire HPLC analysis was provided by Hangzhou Wahaha Group (Hangzhou, China).

### Data source and bioclimatic variables

**Figure 10:**
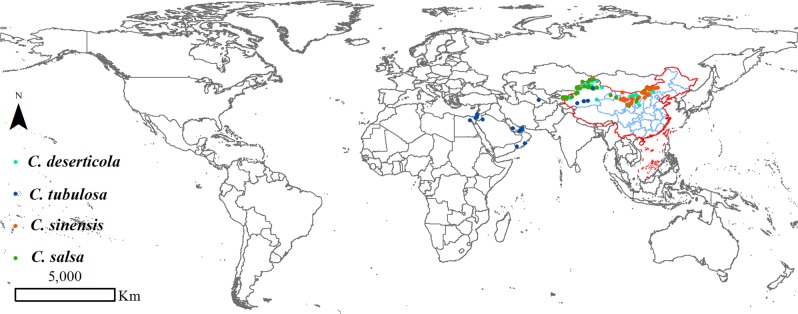
The detailed location distributions of four *Cistanche* species in the world map. (The figure was accomplished by ArcGIS software, version 10.0).

One hundred and forty-six occurrence records of four *Cistanche* plants in the world were collected from databases, including field survey data between 2017 and 2019, the Global Biodiversity Information Facility (http://www.gbif.org), the National Specimen Information Infrastructure (http://www.nsii.org.cn/), and the Chinese Virtual Herbarium (http://www.cvh.org.cn/). Herein, 49 records belong to *C. deserticola*, and *C. tubulosa* has 26 occurrence records in the world. *C. salsa* has 45 data points, and *C. sinensis* has 26 sites, all of which are distributed in China. The detailed location distributions of the four species are shown on the world map and are mapped in Fig. 10. In cases with replicated data points, only one sample was used to reduce sampling bias with regard to environmental conditions. Each data point was converted into World Geodetic System 1984 geographic coordinates by ArcGIS (version 10.0, Environmental Systems Research Institute, Inc. USA)^24^.

Bioclimatic variables are biologically meaningful basal information for the prediction of potential species distribution. Twenty-six bioclimatic variables were downloaded from WorldClim (http://www.worldclim.org), which has average monthly climate data for 1970–2000^25^. All environmental data used in the MaxEnt model were at a 1-km spatial resolution (often referred to as 30 arc-second spatial resolution). Nineteen bioclimatic were coded as bio1-bio19, and the seven remaining factors comprised 7 × 12 variables. For example, variable “Precipitation” contained 12 variables from January to December in one year. The detailed variable information is shown in Table 3. A geographical base map of China was obtained from the National Fundamental Geographic Information System (http://nfgis.nsdi.gov.cn).

**Table 3:** The detailed variables information in present study.

Abbreviation	Variable	Unit
bio01	Annual Mean Temperature	°C
bio02	Mean Diurnal Range (Mean of monthly (max temp - min temp))	°C
bio03	Isothermality (Bio2/Bio7) (* 100)	—
bio04	Temperature Seasonality (standard deviation *100)	C of V
bio05	Max Temperature of Warmest Month	°C
bio06	Min Temperature of Coldest Month	°C
bio07	Temperature Annual Range (BIO5-BIO6)	°C
bio08	Mean Temperature of Wettest Quarter	°C
bio09	Mean Temperature of Driest Quarter	°C
bio10	Mean Temperature of Warmest Quarter	°C
bio11	Mean Temperature of Coldest Quarter	°C
bio12	Annual Precipitation	mm
bio13	Precipitation of Wettest Month	mm
bio14	Precipitation of Driest Month	mm
bio15	Precipitation Seasonality (Coefficient of Variation)	C of V
bio16	Precipitation of Wettest Quarter	mm
bio17	Precipitation of Driest Quarter	mm
bio18	Precipitation of Warmest Quarter	mm
bio19	Precipitation of Coldest Quarter	mm
tmin (01–12)	Minimum Temperature	°C
tmax (01–12)	Maximum Temperature	°C
tavg (01–12)	Average Temperature	°C
pre (01–12)	Precipitation	mm
srad (01–12)	Solar Radiation	kJ·m^−2^·day^−1^
wind (01–12)	Wind Speed	m·s^−1^
vapr (01–12)	Water Vapor Pressure	kPa

### Chromatographic analyses

For each sample, 1 mg of powder was precisely weighed with an electronic balance (Precisa, Switzerland). Then, the sample powder was extracted with 35 ml of 50% methanol solution by an ultrasound-assisted method (180 W, 40 kHz, 30 °C) for 40 min. The cooled extracts were filtered through filter paper after the replenishment of a volatilization solution with 50% methanol solution. Filtration was conducted though a 0.22 μm hydrophobic filter (Millipore, USA) before injection into the autosampler vials. The organic phase (A) was 100% acetonitrile, and the water phase (B) was 0.2% formic acid with a flow rate of 1.0 ml/min. An elution linear gradient was set in accordance with the following scheme: 10% → 15% A (0.00 min → 10 min), 15% → 40% A (10 min → 30 min), 10 min before the next injection. The column temperature was controlled at 27 °C. The filtrate was measured using a Waters 1525 HPLC system (USA) C18 column (4.6 μm, 3.9 × 150 mm) and an evaporative light-scattering detector. The chemical signals were scanned at 330 nm ultraviolet wavelength, and the injection volume was 10 μl.

### Establishment of the MaxEnt model

The MaxEnt method aims to establish a prediction model with a maximum entropy based on existing occurrence records^26,27^. The theory of maximum entropy provides an excellent explanation for approximating an unknown probability distribution^28^. The main principle of the model is the calculation of the maximum entropy of the probability distribution in the target region under the special restraint condition. In light of the calculation results, the model produces a probability distribution of the target species in the research areas. Generally, the environmental variables and occurrence records are restraining conditions. The model continuously overlaps the known sample data for which the probability distribution of the target region increases until its maximum entropy increases to the convergence threshold.

Java-based software (version 3.4.1) was freely downloaded from http://www.cs.princeton.edu/schapire/maxent for habitat suitability simulation. Prior to the model establishment, each variable was converted from the tagged image file format to the action script communication (ASC) format to form the environmental layers. We selected “Auto features” (including Linear, Quadratic, Product and Hinge features) as our model features. A jackknife test was performed to measure the importance of the environmental variables. The random test percentage was set to 30; that is, 70% of the data points were randomly selected as training data, and the remaining 30% occurrence records were the test data. Cross validation was used as the replicated run type. Default options were selected for the other settings.

After using the MaxEnt software, we were able to obtain a file (in ASC format) that reflected the habitat suitability distribution. The file also showed the random data point distribution, which covered the training and test sets with two colors. The file was further transformed into raster data to improve the interpretation and calculation of the suitable location. Furthermore, the raster file was reclassified into four classes comprised of very low suitable class or a non-suitable class (4, <0.2), low suitable class (3, threshold between [0.2, 0.4]), moderately suitable class (2, [0.4, 0.6]) and high habitat suitability class (1, [0.6, 1])^29–31^. The number indicates the class number in the final figure. A commonly used threshold of 0.4 was used for the class division^30^. The format transformation and calculation of suitable areas were completed by ArcToolbox in ArcGIS software.

The receiver operating characteristic (ROC) curve and area under the curve (AUC) were used to analyze the fitting capability and to comprehensively evaluate the performance of the well-established model. In general, the closer the top left corner the ROC curve is to 1, the more robust the model. A high AUC value implies a superior model performance, which is not affected by the choice of the threshold^32^. The normal value of the latter parameter is between 0.5 and 1. In accordance with the range of parameters, the MaxEnt model performance was categorized into six standards comprising a pure guess (AUC = 0.5), fail (0.5 < AUC ≤ 0.6), poor (0.6 < AUC ≤ 0.7), fair (0.7 < AUC ≤ 0.8), good (0.8 < AUC ≤ 0.9), and excellent (0.9 < AUC ≤ 1). The parameter range indicates that a value close to 1 signifies good model discrimination performance^33^.

### Statistical method

Data were subjected to analysis of variance using SPSS software (version 21.0, IBM Corp., Armonk, USA) to investigate the content difference of chemical components from different geographical origins and species. The difference was determined using the least significant difference test at 5% probability (*P* ≤ 0.05).

## Supplementary Information

Supplementary Information
