# Supplementary material for: Chemical Diversity and Prediction of Potential Cultivation Areas of Cistanche Herbs
Source: Sci Rep. 2019 Dec 24;9:19737. doi: 10.1038/s41598-019-56379-x (PMC6930302; doi:10.1038/s41598-019-56379-x)

Chemical Diversity and Prediction of Potential Cultivation Areas of *Cistanche* Herbs

Ye Wang[1], Li Zhang[2], Zhixia Du[1,3], Jin Pei[3*], Linfang Huang[1*]

[1] Institute of Medicinal Plant Development, Chinese Academy of Medical Sciences, Peking Union Medical College, Engineering Research Center of Chinese Medicine Resource, Ministry of Education, Key Research Laboratory of Traditional Chinese Medicine Resources Protection, Administration of Traditional Chinese Medicine, National Administration of Traditional Chinese Medicine, Beijing 100193, China

[2] College of Science, Sichuan Agricultural University, Ya'an 625000, China

[3] College Pharmacy, Chengdu University of Traditional Chinese Medicine, Chengdu 611137, China

[*]**Corresponding authors**:

Jin Pei: peixjin@163.com

Linfang Huang: linfanghuang416@163.com and lfhuang@implad.ac.cn

Table S1 Important variables based on percent contribution and permutation contribution in *C. deserticola.*

Variable	Percent contribution	Permutation importance	Variable	Percent contribution	Permutation importance
bio02	0.2	0.5	srad11	0.1	2.2
bio08	0	1.6	srad12	0.1	0
bio09	8.2	23.2	tavg03	6.6	0
bio12	0	0.3	tavg05	1.5	0.4
bio14	0.1	0.1	tmax01	0.5	29.7
bio15	0.1	0	tmax02	10	18.3
bio16	2.8	0.1	tmax03	0.1	0.1
bio19	7.6	0	tmax04	0.1	0
pre01	11.6	0.6	tmax05	0.3	0
pre03	0.8	0.7	tmax11	0.1	3.2
pre05	1.1	1.4	tmax12	12.6	0
pre06	1.5	0	tmin04	0	0.1
pre07	0.1	0	tmin11	0.6	0
pre09	4	0	vapr04	0.5	2.6
pre10	0.4	0	vapr07	0.3	1.6
srad02	0	0.3	vapr08	0.1	0.4
srad03	0	0.1	vapr12	0.1	0
srad05	0.4	3.5	wind01	6.3	3.3
srad08	13.2	4.8	wind10	0.5	1.1
srad09	7.5	0			

Table S2 Important variables based on percent contribution and permutation contribution in *C. tubulosa*.

Variable	Percent contribution	Permutation importance	Variable	Percent contribution	Permutation importance
bio03	0	1	srad05	20.6	0
bio05	0.1	0.5	srad06	3.3	2.1
bio16	1.5	5.3	srad09	4.1	0
bio17	0.5	0.2	tavg06	0.7	0.5
pre01	2.4	4.8	tavg07	1.3	0
pre02	0.9	0	tmax06	0.1	0.1
pre03	0.2	0	tmin09	0.2	0
pre04	0.9	4.6	vapr01	0.2	0
pre05	1	1.9	vapr06	0.6	17.8
pre07	0.3	0	vapr07	0.9	0
pre08	14.9	50	vapr08	13.4	0
pre09	20.9	1.4	vapr09	1.9	0
pre10	5.1	6.8	wind07	0.2	2.9
pre11	0.5	0	wind10	2.2	0
srad01	1	0			

Table S3 Important variables based on percent contribution and permutation contribution in *C. salsa*.

Variable	Percent contribution	Permutation importance	Variable	Percent contribution	Permutation importance
bio08	0.1	0.7	srad06	0.8	0.3
bio09	2.5	6.3	srad07	0.1	0
bio12	1.2	2	srad08	12.1	3.2
bio15	0.3	0	srad09	2.2	0.6
bio16	5.1	0.9	srad11	0.2	0
bio19	11.2	0	srad12	2.8	0
pre01	10.9	2.3	tavg03	3.4	0
pre02	0.7	1	tmax01	3.5	0.2
pre03	1	0.2	tmax02	29.3	63.2
pre04	0	0.2	tmax11	0.1	0
pre05	1.8	0.1	tmin04	0.3	14.3
pre06	0.3	0	vapr07	0.2	0
pre09	1.1	0	vapr08	0.1	1.4
pre11	2.6	0.2	wind01	4.6	0.1
pre12	0.6	0.6	wind02	0.1	0.1
srad04	0.2	0.9	wind10	0.6	1.4

Table S4 Important variables based on percent contribution and permutation contribution in *C. sinensis*.

Variable	Percent contribution	Permutation importance	Variable	Percent contribution	Permutation importance
bio03	0.1	1.7	tavg06	0.1	0
bio14	5.4	0	tavg08	0.9	0
bio17	0	0	tavg11	2.6	0
bio19	23.3	0	tmax02	26	0
pre01	0.3	0	tmax04	0.3	23.9
pre02	0.1	0	tmax10	0.1	0
pre04	0.5	0	tmax11	0.2	11.7
pre05	0.2	0	tmin04	0.3	0
pre06	3.4	0	tmin06	0.1	15
pre09	1.5	0.3	tmin10	0.7	0
pre10	7.1	0.2	vapr01	0	1.8
pre12	15.5	0.2	vapr04	0.3	25.2
srad03	0.9	8.6	vapr05	0.4	0
srad04	1.7	0	vapr06	0.1	0
srad08	2.9	2.1	vapr07	0.3	0
srad12	3.7	0	vapr12	0.2	0
tavg03	0.5	0	wind08	0.1	5.9
tavg04	0.1	3.4			

Figure S1 Results of the jackknife test in *C. deserticola* model.

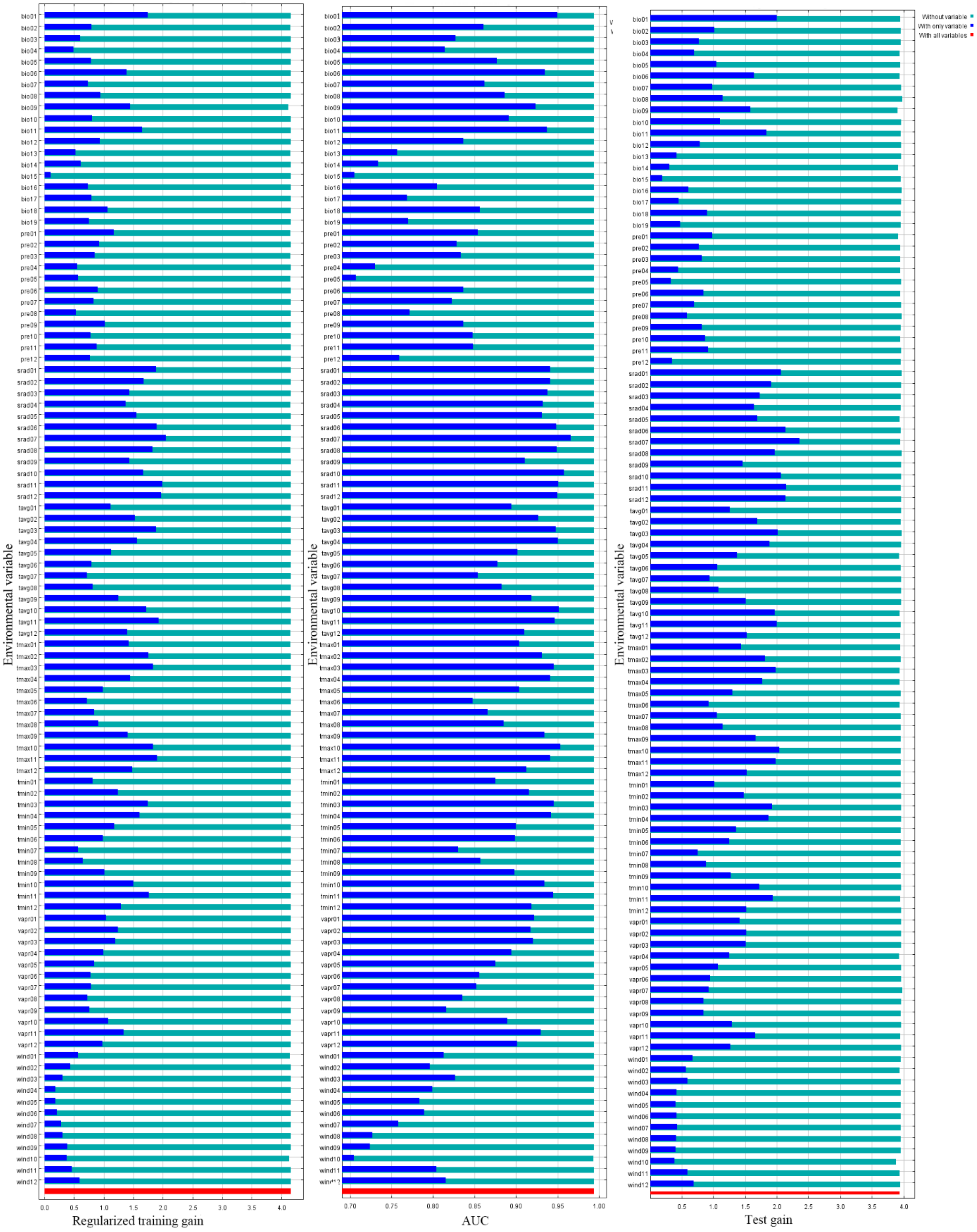

Figure S2 Results of the jackknife test in *C. tubulosa* model.

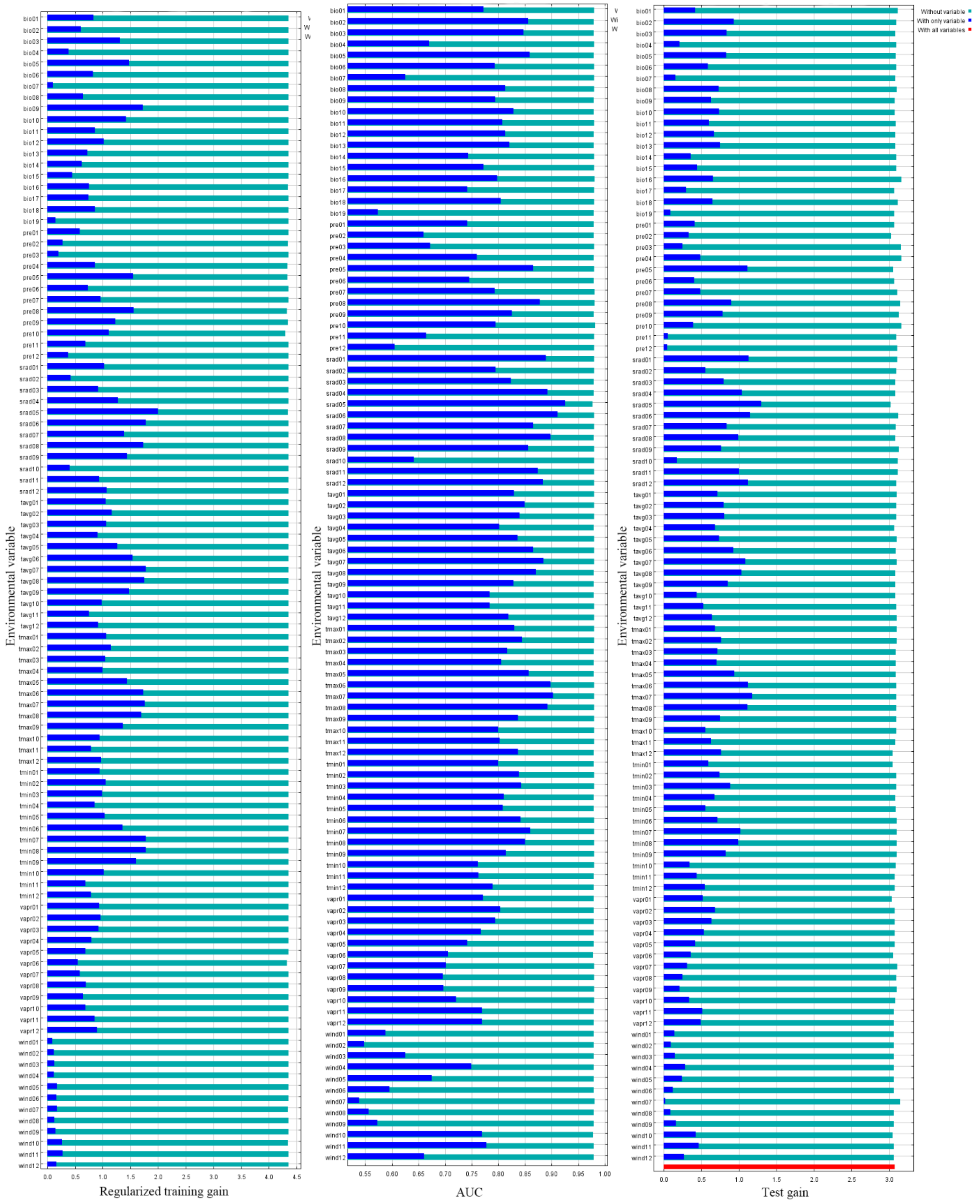

Figure S3 Results of the jackknife test in *C. salsa* model.

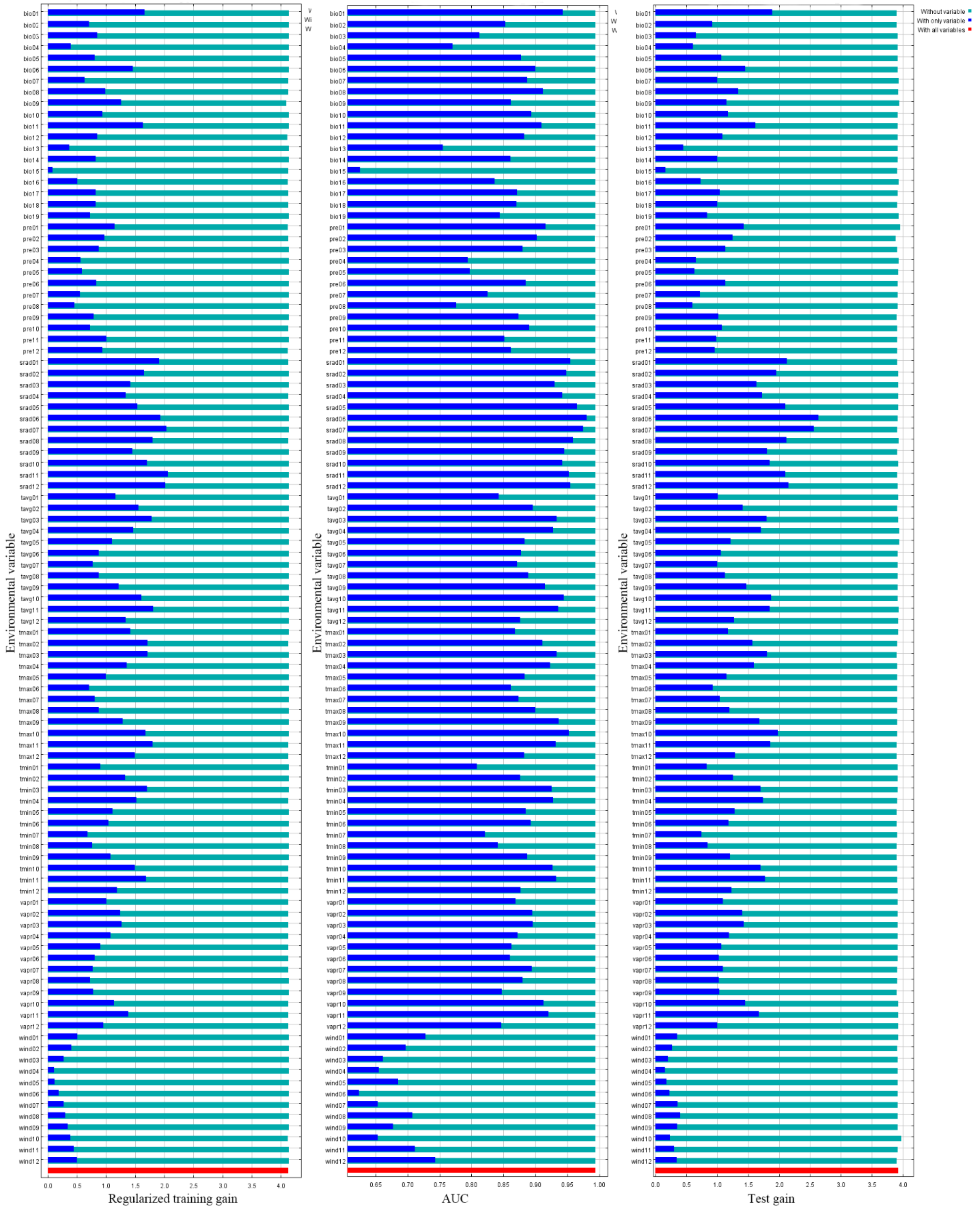

Figure S4 Results of the jackknife test in *C. sinensis* model.

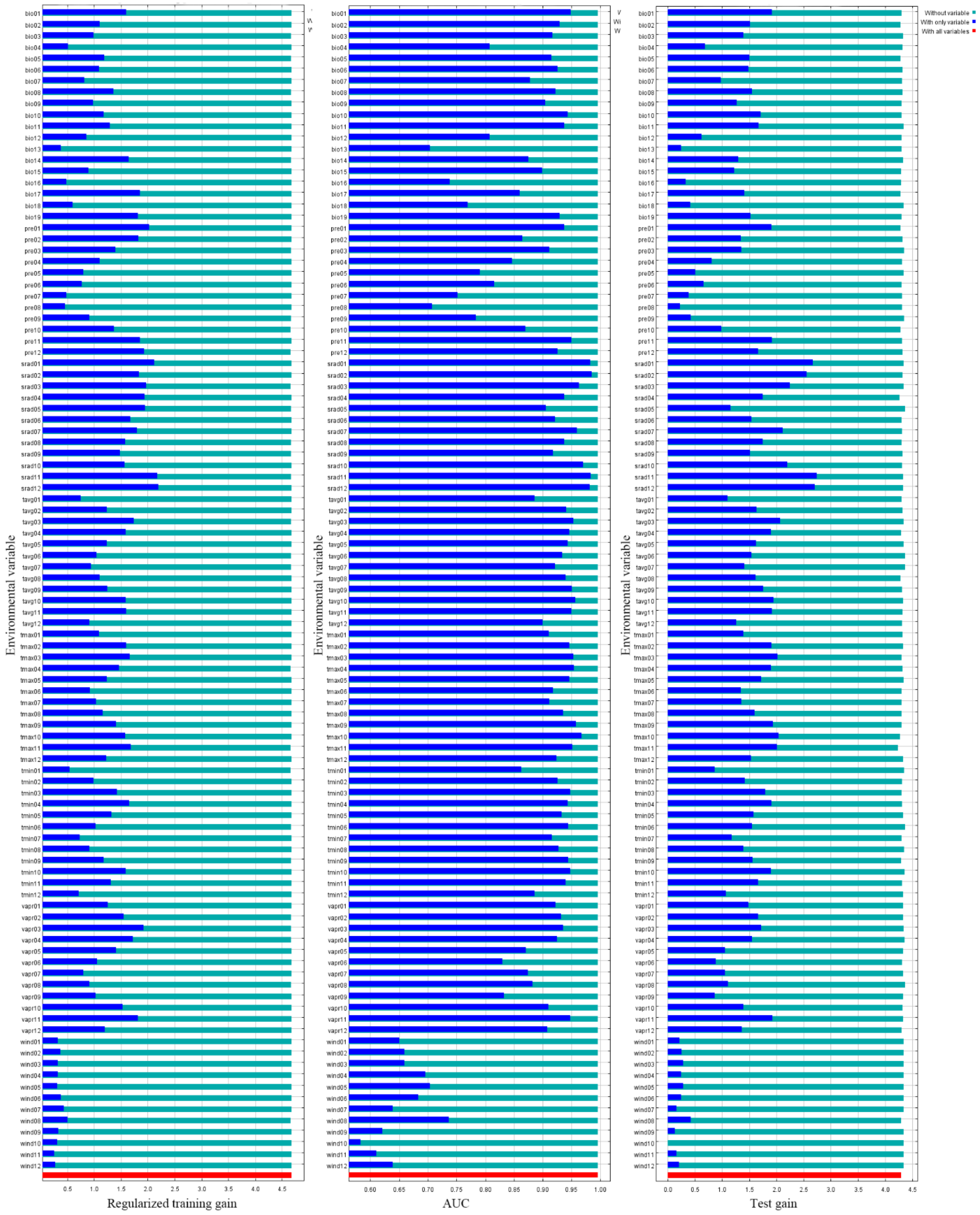

Figure S5 Variables' response results to suitability for *C. deserticola*.

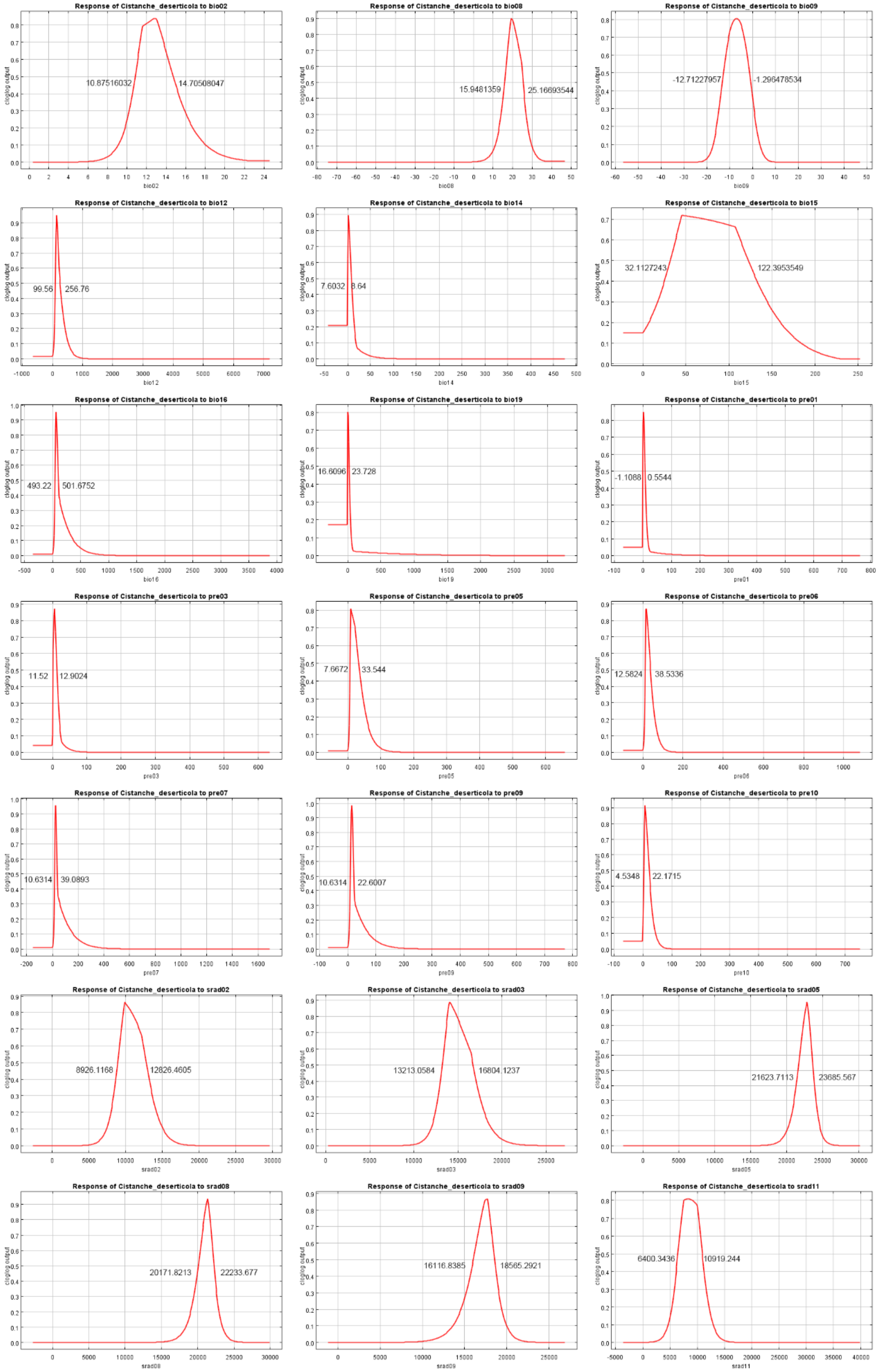

Figure S5 Continued…

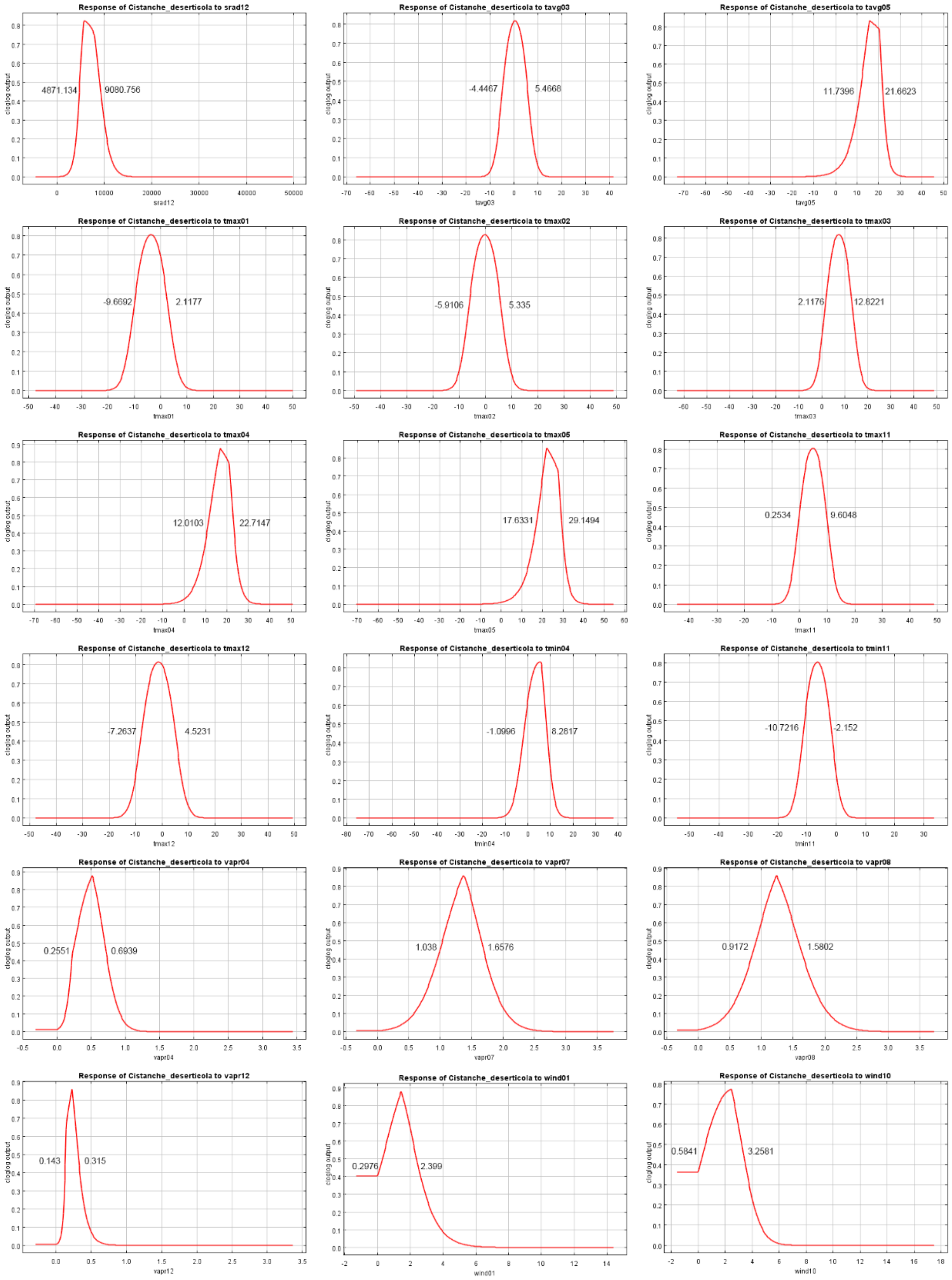

Figure S6 Variables' response results to suitability for *C. tubulosa*.

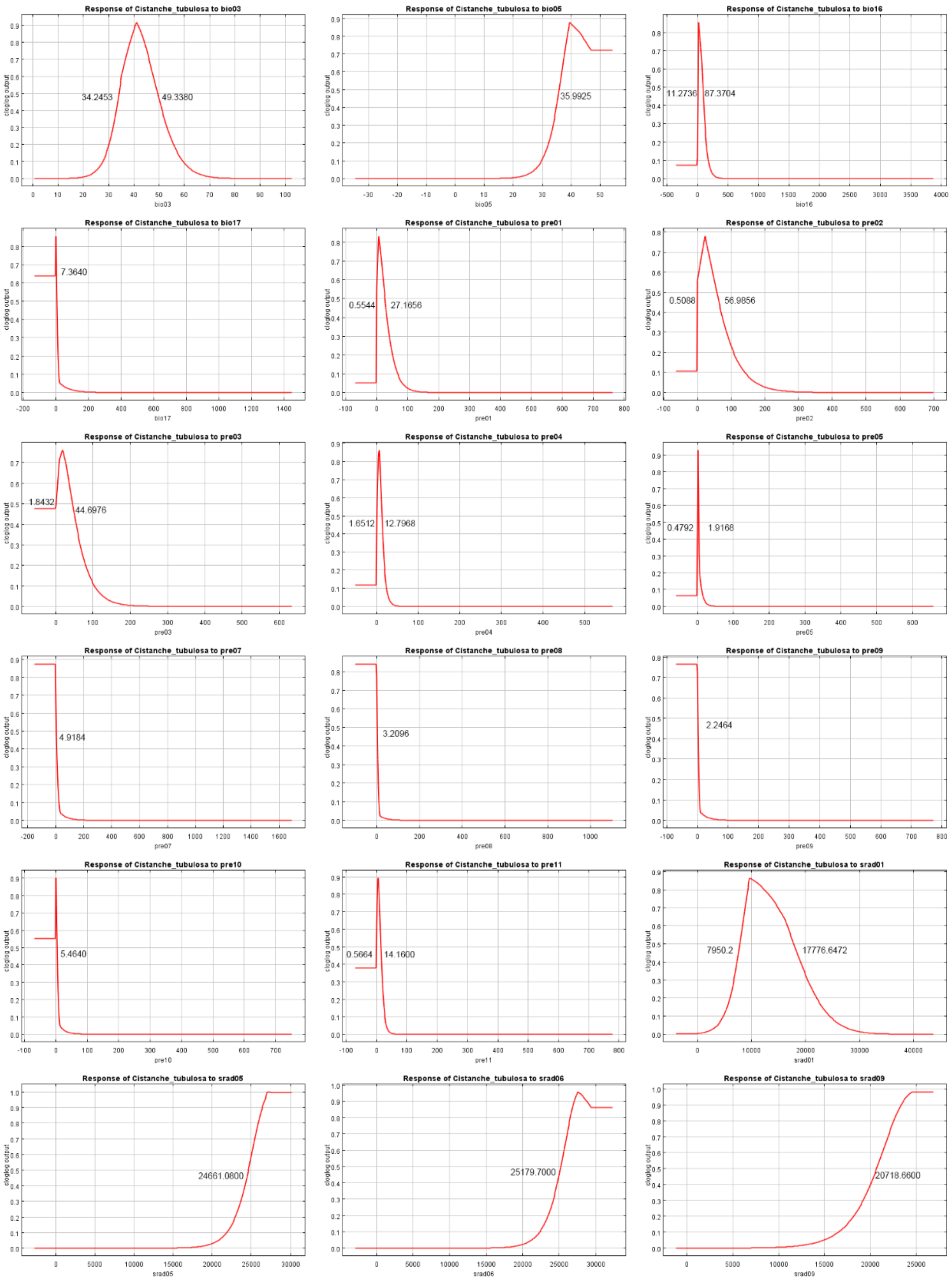

Figure S6 Continue…

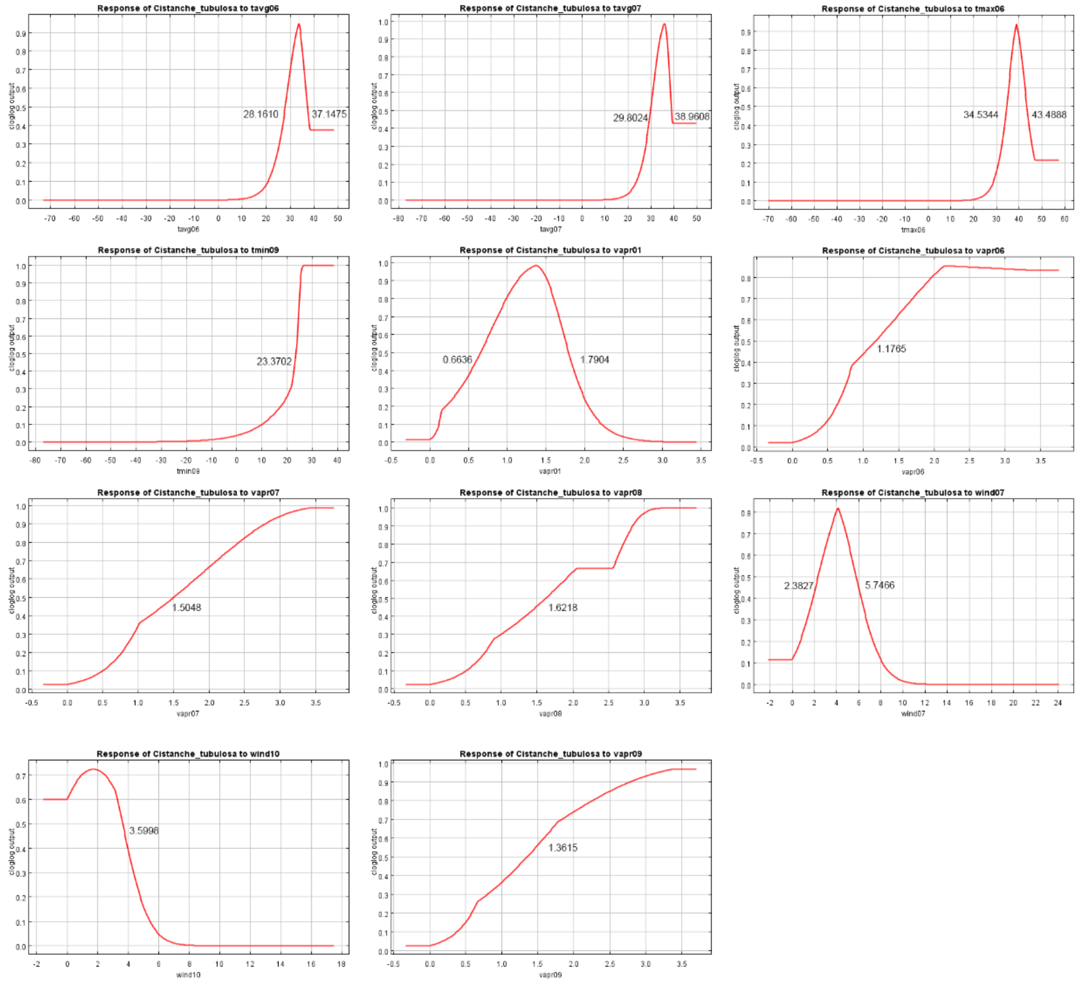

Figure S7 Variables' response results to suitability for *C. salsa*.

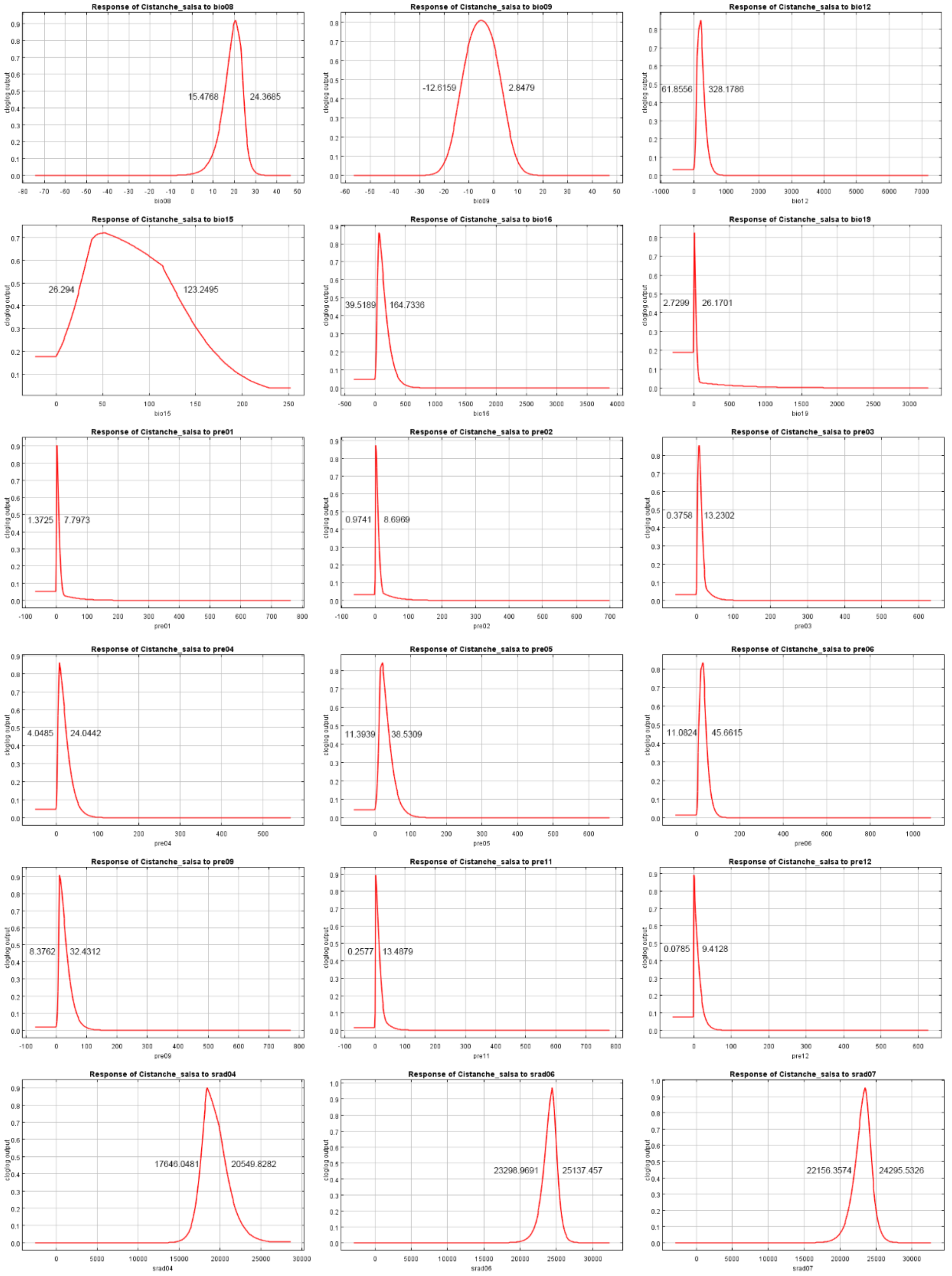

Figure S7 Continued…

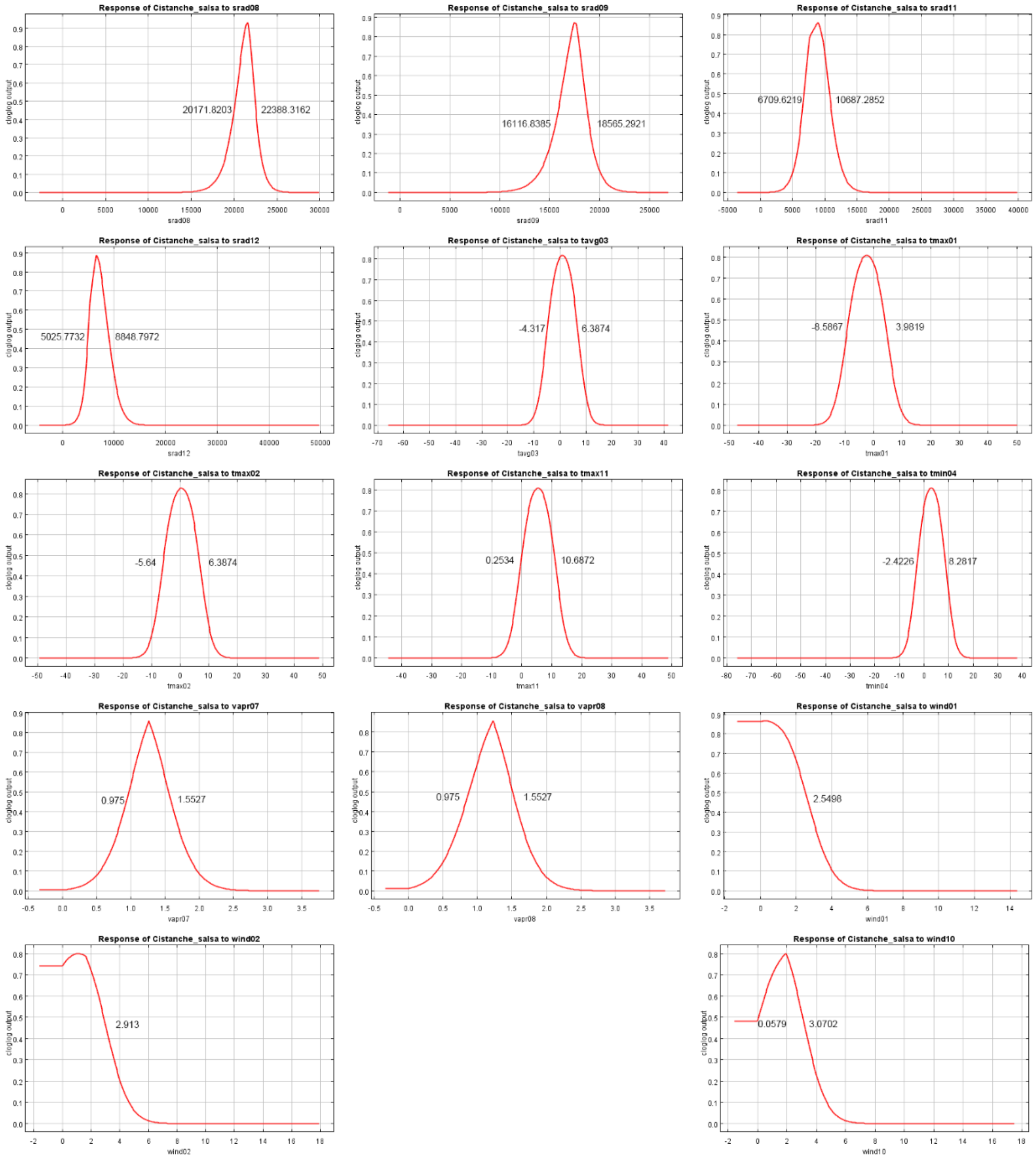

Figure S8 Variables' response results to suitability for *C. sinensis*.

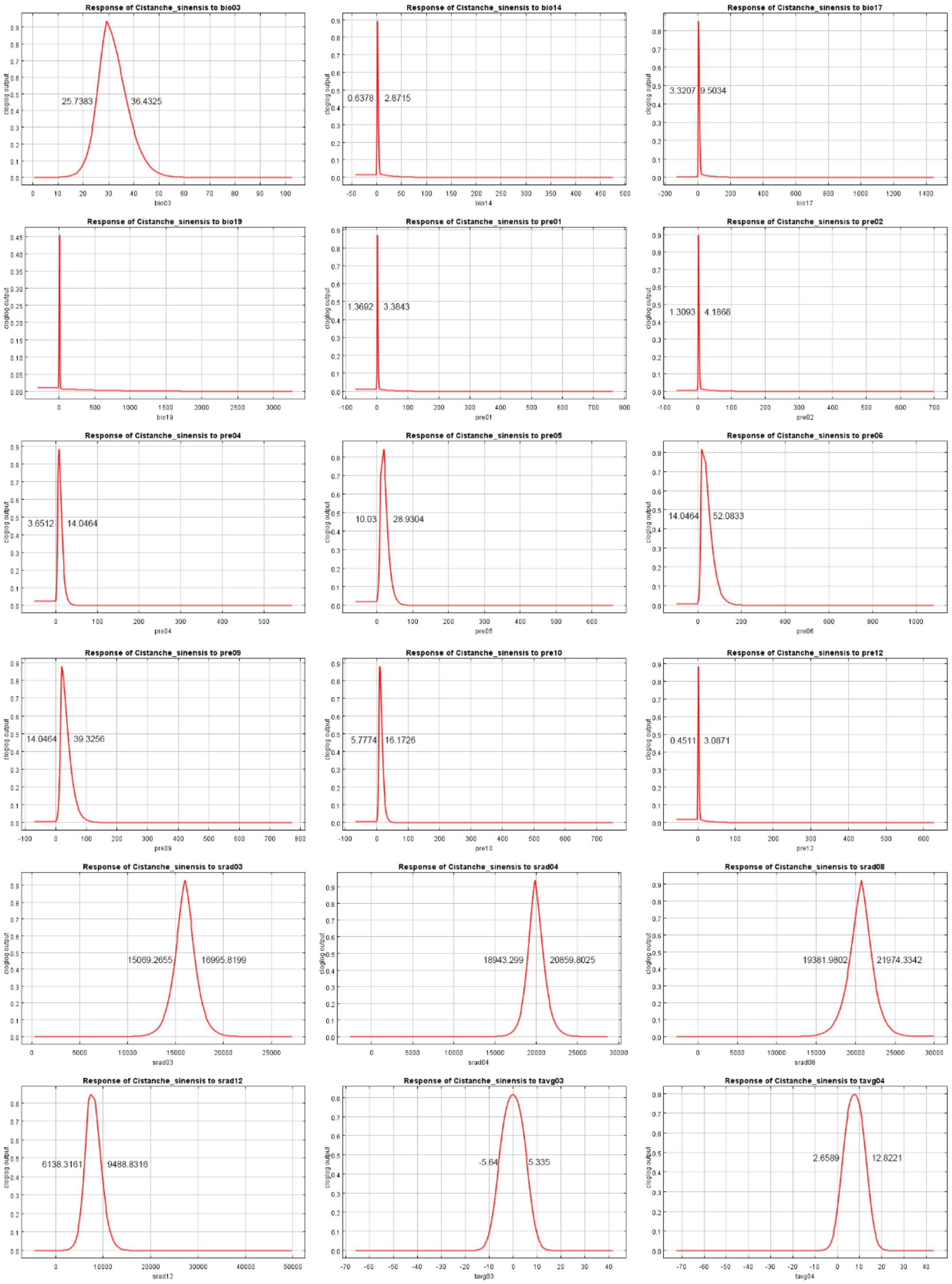

Figure S8 Continued…

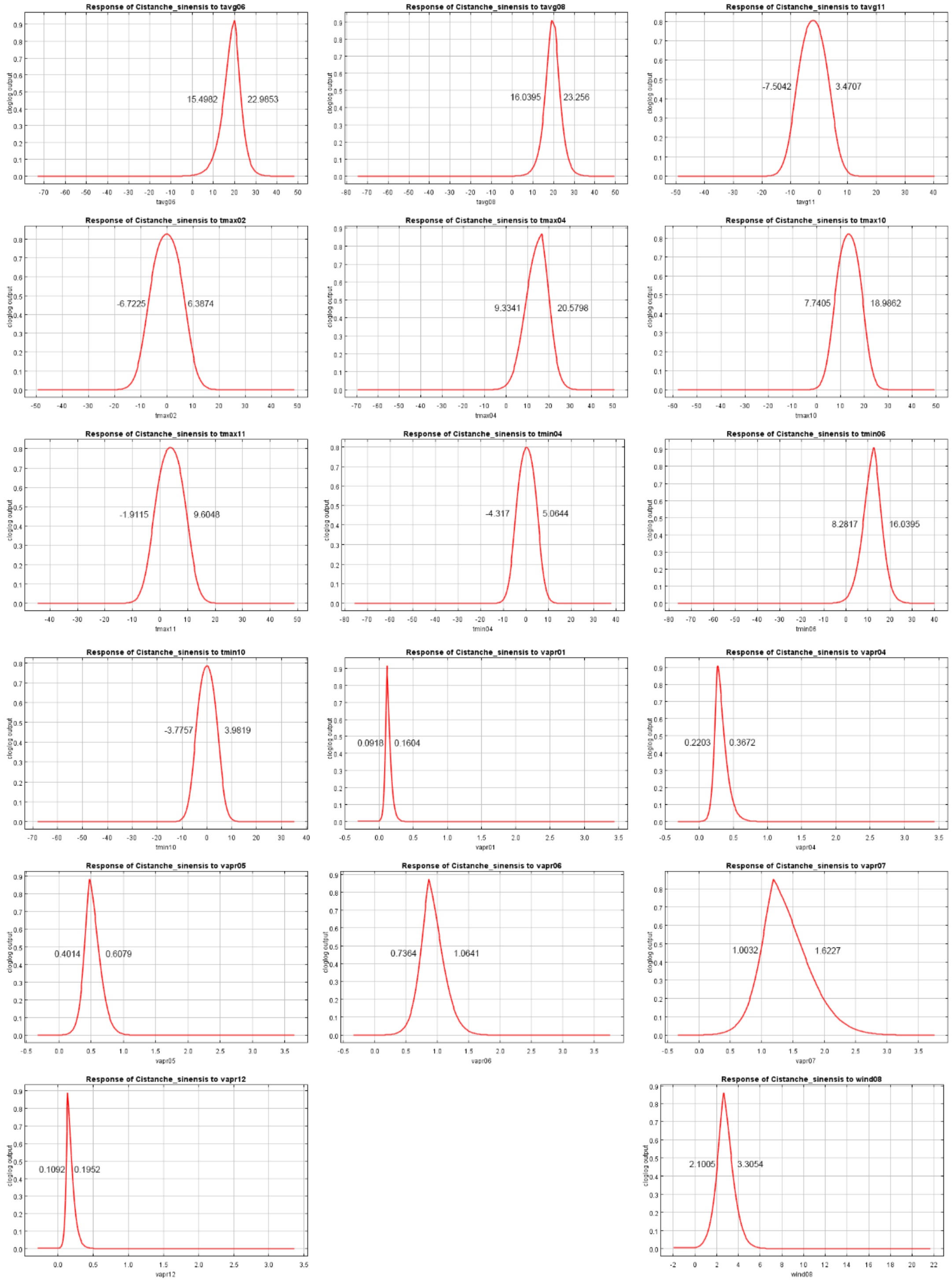

Supplement: Supplementary file 1 — Supplementary Information [file 41598_2019_56379_MOESM1_ESM.pdf]
